# Canonical Cortical Circuit Model Explains Rivalry, Intermittent Rivalry, and Rivalry Memory

**DOI:** 10.1371/journal.pcbi.1004903

**Published:** 2016-05-03

**Authors:** Shashaank Vattikuti, Phyllis Thangaraj, Hua W. Xie, Stephen J. Gotts, Alex Martin, Carson C. Chow

**Affiliations:** 1 Mathematical Biology Section, Laboratory of Biological Modeling, National Institutes of Diabetes and Digestive and Kidney Disease, National Institutes of Health, Bethesda, Maryland, United States of America; 2 Cognitive Neuropsychology Section, Laboratory of Brain and Cognition, National Institute of Mental Health, National Institutes of Health, Bethesda, Maryland, United States of America; University College London, UNITED KINGDOM

## Abstract

It has been shown that the same canonical cortical circuit model with mutual inhibition and a fatigue process can explain perceptual rivalry and other neurophysiological responses to a range of static stimuli. However, it has been proposed that this model cannot explain responses to dynamic inputs such as found in intermittent rivalry and rivalry memory, where maintenance of a percept when the stimulus is absent is required. This challenges the universality of the basic canonical cortical circuit. Here, we show that by including an overlooked realistic small nonspecific background neural activity, the same basic model can reproduce intermittent rivalry and rivalry memory without compromising static rivalry and other cortical phenomena. The background activity induces a mutual-inhibition mechanism for short-term memory, which is robust to noise and where fine-tuning of recurrent excitation or inclusion of sub-threshold currents or synaptic facilitation is unnecessary. We prove existence conditions for the mechanism and show that it can explain experimental results from the quartet apparent motion illusion, which is a prototypical intermittent rivalry stimulus.

## Author Summary

When the brain is presented with an ambiguous stimulus like the Necker cube or what is known as the quartet illusion, the perception will alternate or rival between the possible interpretations. There are neurons in the brain whose activity is correlated with the perception and not the stimulus. Hence, perceptual rivalry provides a unique probe of cortical function and could possibly serve as a diagnostic tool for cognitive disorders such as autism. A mathematical model based on the known biology of the brain has been developed to account for perceptual rivalry when the stimulus is static. The basic model also accounts for other neural responses to stimuli that do not elicit rivalry. However, these models cannot explain illusions where the stimulus is intermittently switched on and off and the same perception returns after an off period because there is no built-in mechanism to hold the memory. Here, we show that the inclusion of experimentally observed low-level background neural activity is sufficient to explain rivalry for static inputs, and rivalry for intermittent inputs. We validate the model with new experiments.

## Introduction

Perceptual rivalry is the subjective experience of alternations between competing percepts when an individual is presented with an ambiguous stimulus. It has been suggested that rivalry between neurons may be a ubiquitous property and competition between neural representations occurs throughout the brain [1]. Evidence for this includes: 1) neural correlates of visual rivalry found at multiple regions of visual processing (e.g., V1, V4, MT, IT) [2–5], 2) most sensory modalities exhibit rivalry suggesting similar biophysical processes [6], 3) brain regions that do not receive specific sensory inputs, such as the inferior frontal and parietal lobe, exhibit activity that is correlated with perceptual switches [7], and 4) independent rivalry can occur at different spatial locations, with different senses, and under different modalities [6,8–10]. Rivalry provides a unique window into cortical function because it directly accesses an internal computation elicited by but distinct from an external sensory input. Many experiments on rivalry utilize stimuli that are always in view although possibly moving. We refer to these situations as *static rivalry*. In *intermittent rivalry*, the stimulus presentation is periodically removed while the perception alternates with a longer period. *Rivalry memory* refers to the return of the last dominant percept after an extended time duration without stimulus.

Computational and mathematical work has shown that a neurophysiologically-constrained cortical model whose primary features are mutual inhibition between pools of neurons and a fatigue process can reproduce the basic experimental properties of static rivalry, winner-take-all behavior, normalization, and decaying oscillations induced by distractors under different input conditions [11–14]. These findings support the universality of a simple neuronal model that can be used to explain a myriad of basic cortical behaviors and as such is a candidate canonical circuit for simple cognitive functions (e.g. see [15–17] for discussion of such functions). A canonical circuit is universal in the sense that it does not imply a single function but can exhibit multiple operating regimes under a change in parameter values (including just a change in the type of stimulus). The biophysical dynamics can be inferred from behavioral observations such as rivalry, decision-making, working memory, and contrast-sensitivity [1,6,13,18] making them potential clinical endophenotypes (simple biomarkers associated with and possibly underlying a more complex trait of interest). As such, rivalry has been suggested as a diagnostic tool for probing cognitive dysfunction such as in autism, bipolar disorder, and major depressive disorder [19–21].

The canonical circuit and similar models have been validated against a set of nontrivial observations in static rivalry including Levelt’s propositions [11,22–26]. Although mutual inhibition acting as a positive feedback has been proposed as a memory mechanism in other contexts [27], it has been argued [22,28] that a circuit with mutual inhibition and fatigue cannot reproduce intermittent rivalry nor rivalry memory because presumably there is no mutual inhibition when the stimulus is absent and the fatigue process would give the opposite result of what is observed, favoring the suppressed percept (i.e. masking not priming). The question then is whether a single circuit model can account for all of the aforementioned behaviors (including non-rivalry dynamics) or whether independent or additional circuits are required to explain all the phenomena.

Here, we show that the same cortical circuit model can also be applied to time-varying inputs in intermittent rivalry and rivalry memory. We explore various possible mechanisms and show that just the inclusion of a previously neglected phenomenon—namely low, nonspecific, background activity—is sufficient to provide a unified account of static rivalry, intermittent rivalry, rivalry memory and all previously accounted for phenomena. The background activity at arbitrarily low values can provide sufficient drive to the neurons such that the memory can be held by mutual inhibition alone and withstand the effects of fatigue. The memory persists over a wide range of parameter values, is robust to noise, and does not require the inclusion of another process. The memory is topological in the sense that the total number of memory states is invariant to a range of changes to the input and network properties. Importantly this memory preserves all of the previously known behaviors of the basic model since the background activity does not play a role under the static conditions. This is in contrast to recurrent excitation models of short-term memory where neural activity is bistable between an active and inactive state, which is generally antagonistic to rivalry [29], may conflict with local balanced states [30], and relies on fine-tuning [31].

The circuit model can reproduce two main features of rivalry memory and intermittent rivalry that we observed in the ambiguous quartet illusion: 1) a dynamic Levelt’s fourth proposition (*dynamic L4*), where the dominance duration increases with increasing stimuli presentation intervals and 2) *habituation*, where the initial percept durations (epochs) decrease upon repeated stimulus presentations until a steady state is reached after several epochs. Dynamic L4 has been shown in prior work with other intermittent paradigms [32]. Habituation has also been reported for rivalry; although, not necessarily intermittent rivalry [33–35]. We provide a theoretical explanation for these behaviors. The theory suggests that for the observed habituation to occur, local fatigue such as spike-frequency adaptation or recurrent synaptic depression is the dominant form of fatigue rather than synaptic depression between pools, and that the interval between pulses is shorter than the fatigue time constant.

## Results

### Experiment Results

**Fig 1:**
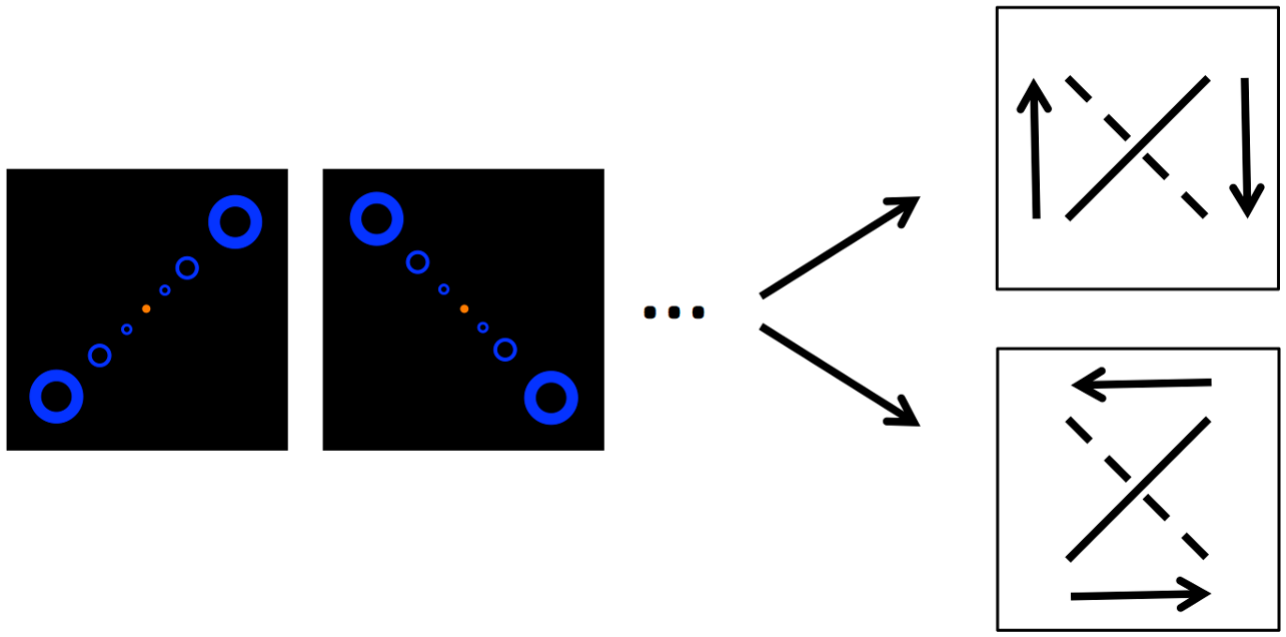
Quartet illusion. We presented a variant of the quartet consisting of nested quartets. A single quartet frame consists of two dots located at opposing corners of a square, across the diagonal. In the next frame the other set of opposing dots is shown. A single transition between frames is perceived as either a motion with the dots moving vertically together as an illusory bar rotating clockwise (top right frame), or horizontally as a bar rotating counterclockwise (bottom right frame).

**Fig 2:**
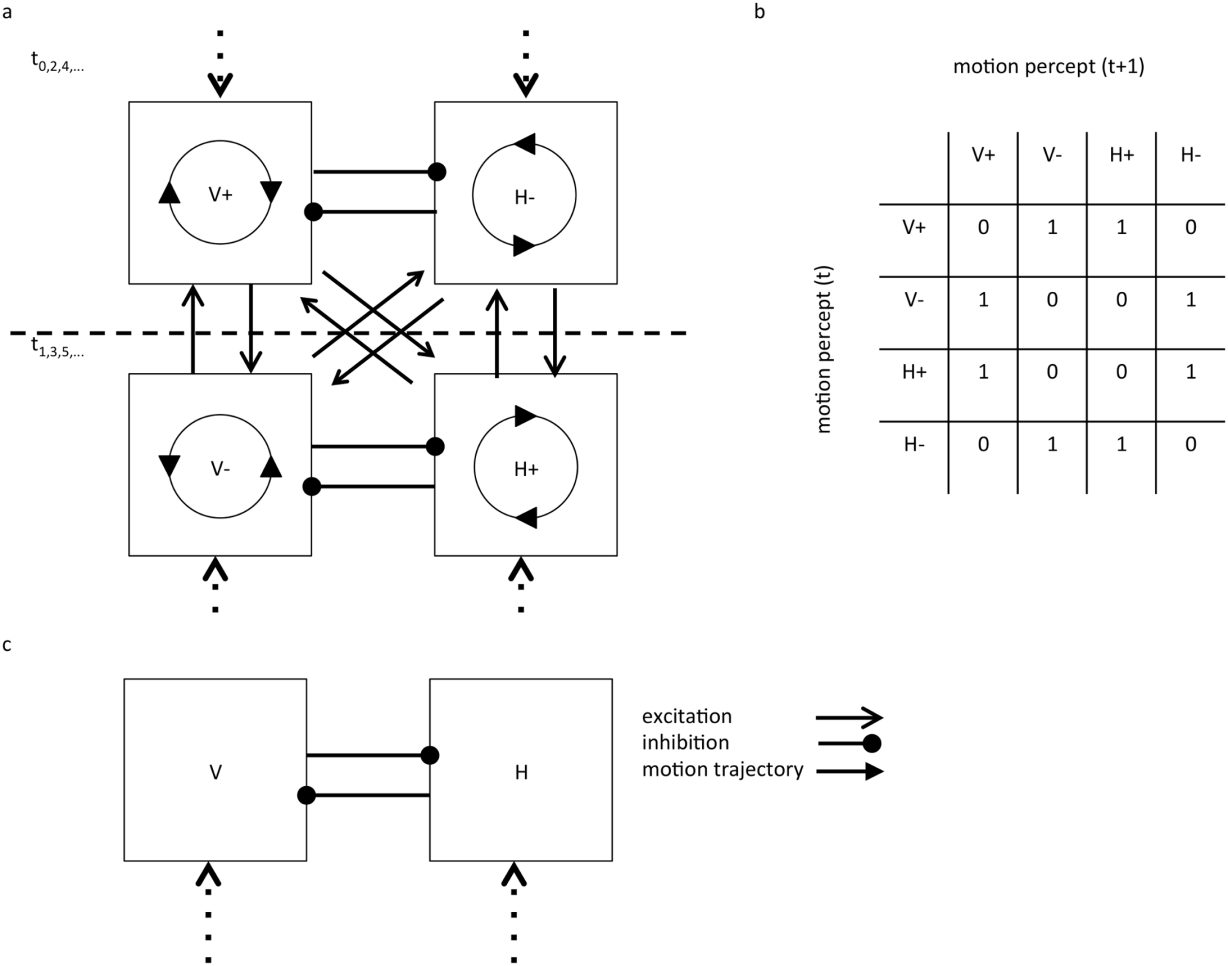
Paths of motion and neural network for the quartet illusion. **(a)** Each frame transition of the quartet illusion (t_0_, t_1_, t_2_,…) induces an ambiguous percept of one of two possible motions characterized by an orientation—horizontal (H) or vertical (V)—and a direction of rotation—clockwise (+) or counter-clockwise (-). During a frame transition there is competition (mutual inhibition) between the two possible perceived motions—V+ competes with H- and V- competes with H+. In the illusion, once an orientation becomes dominant, there is an oscillation in direction on each frame presentation, e.g. V+, V-, V+, etc. Eventually, the other orientation becomes dominant and the oscillations in direction will continue, e.g. H+, H-, H+, etc. The rivalry refers to the alternation between orientations. **(b)** A table of the allowed transitions due to the symmetries in the illusion. Each row indicates the perceived motion at the current transition and each column the perceived motion at the next transition; a value of one in a column indicates a possible path. **(c)** The circuit can be reduced by averaging over the fast direction oscillations into a two pool circuit with mutual inhibition between competing neuronal pools representing H and V orientations with an intermittent drive (dashed lines).

We informed our model with experiments on the ambiguous quartet illusion, which is a prototypical example of intermittent rivalry and has the advantage of naturally incorporating a periodic stimulus (see Fig 1). The inputs are time dependent and the perceived motion only occurs when the frame presentation (input) is switched from one parity (e.g. dots in upper-right and lower-left corners (UR-LL)) to the other (e.g. lower-right and upper-left corners (LR-UL)). The motion is ambiguous because the transition has two possible interpretations. For example, in the transition from UR-LL to LR-UL, the dot located at UR could be interpreted as “sliding” vertically down to LR, while the dot at LL simultaneously slides up to UL. The alternative interpretation is that the dot at UR slides horizontally leftwards to UL, while the other dot at LL slides horizontally rightward to LR. If one imagines a bar connecting the dots then the vertical interpretation corresponds to a bar rotating clockwise from 45° to –45° (like a seesaw) while the horizontal interpretation corresponds to the stick rotating counterclockwise from 45° to 135°. Hence, the perceived motion in the quartet illusion is characterized by two degrees of freedom—orientation and direction. Orientation refers to whether the dot motion is aligned along the vertical (V) or horizontal (H) axis and direction refers to whether the motion is clockwise (+) or counterclockwise (-) (see Fig 2a).

**Fig 3:**
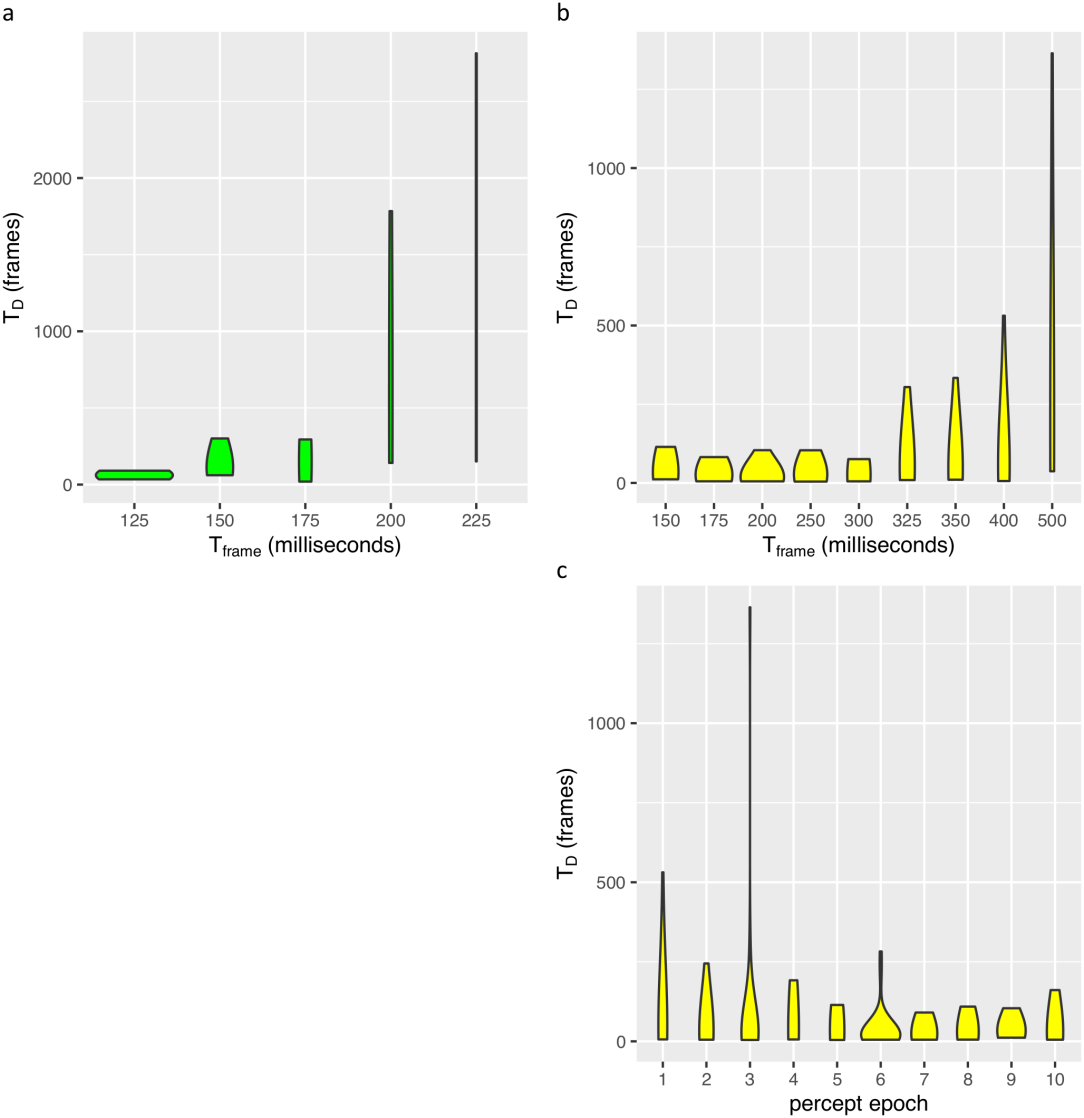
Dynamic L4 and habituation. Violin plots of percept duration (T_D_ in movie frames) versus the interval between changes in frame (T_frame_) for author-subjects A11 **(a)** and A10 **(b)** and versus percept epochs (order of reported percepts) for A10 **(c)**. The width of the violin plot represents the probability distribution for a dominance duration. **(a-b)** Increased T_D_ with T_frame_ (dynamic L4). **(c)** Decreased T_D_ across percept epochs (habituation) for pooled T_frame_ data.

We presented the illusion to subjects with different frame presentation intervals (T_frame_). We examined dynamic L4 by testing if the perception dominance duration (T_D_) (inverse of the percept alternation rate) was correlated with T_frame_. In our first experiment we tested two subjects across a wide range of T_frame_ inputs. The subjects showed a dramatic response to the stimulus parameter, which occurred at different ranges for each subject (different sensitivities). In Fig 3a and 3b we plotted the T_D_ distributions to give the qualitative picture. We also observed some evidence of a habituation effect (Fig 3c), where the dominance durations decreased to a steady state value.

To verify the phenomenon we recorded dominance durations on a group of 16 hypothesis-naive subjects. We probed the system over a range of T_frame_: 300, 350, 400, and 450 milliseconds (minimum chosen to avoid photosensitive epilepsy). Individuals were told to report on any change of motion and were trained on biased H and V motion stimuli prior to the quartet stimulus (see Methods). From our post-task survey we found that all individuals observed oscillating V motion (seesaw) and H motion (seesaw rotated by 90 degrees). For a majority of subjects, these were the only reported percepts but a subset occasionally observed rotation (H, V, H,…). There were also infrequent reports of the dots disappearing or changing size and the dots moving toward and away from the subject (three-dimensional motion). The stimulus used in the experiments can be seen in S1 Movie.

**Fig 4:**
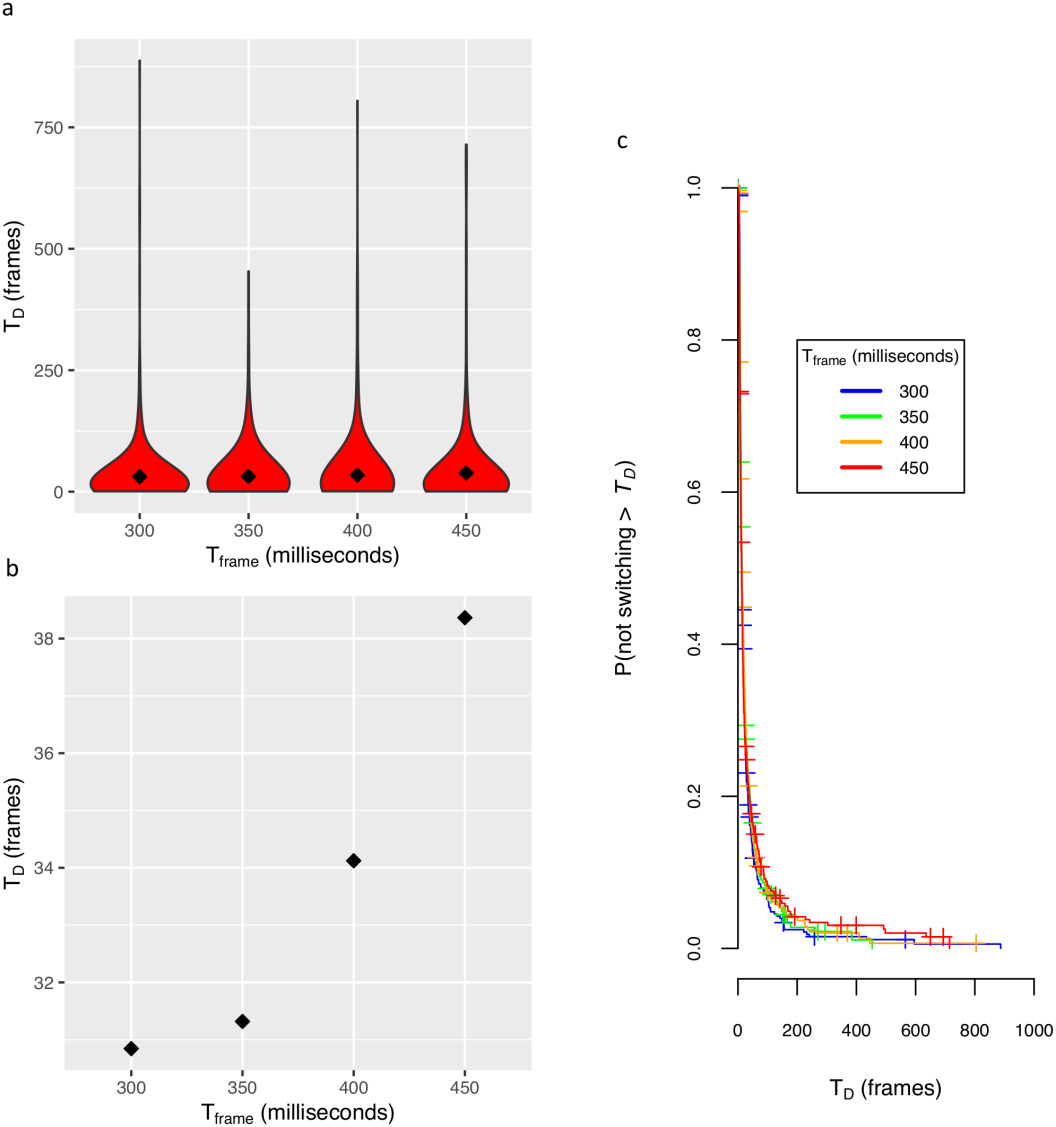
Dynamic L4 survival analysis for pooled hypothesis-naive subject data. **(a)** Violin plots with means (diamonds) for T_D_ versus T_frame_. T_D_ is the reported uninterrupted duration of either horizontal or vertical motion (or for a few subjects rotational motion), in units of the number of presented movie frames. T_frame_ is the interval between movie frames. **(b)** Mean of the pooled data. **(c)** T_frame_ T_D_-survival plots. There is a small but significant difference in survival probabilities.

To test for the presence of dynamic L4, we analyzed the pooled data across subjects and across percept-types (H, V, rotation). We observed a weak effect across subjects. The qualitative results are shown by the T_D_ distributions and their means (see Fig 4a and 4b). We quantified the relationship with a Cox proportional hazards mixed effects model. The statistical model treats the dominance time as the survival time for a percept and evaluates the effects of factors on the survival probability (see Fig 4c). There was a significant but weak positive relationship of T_D_ with T_frame_ with *P =* 2 × 10^−4^ (Wald test using 1,322 samples and 1,266 recorded switch events) and hazard ratio of 0.996 (s.e. 0.001). We used these preliminary findings together with previously reported effects for another rivalrous stimulus [32] as support for the dynamic L4 constraint.

**Fig 5:**
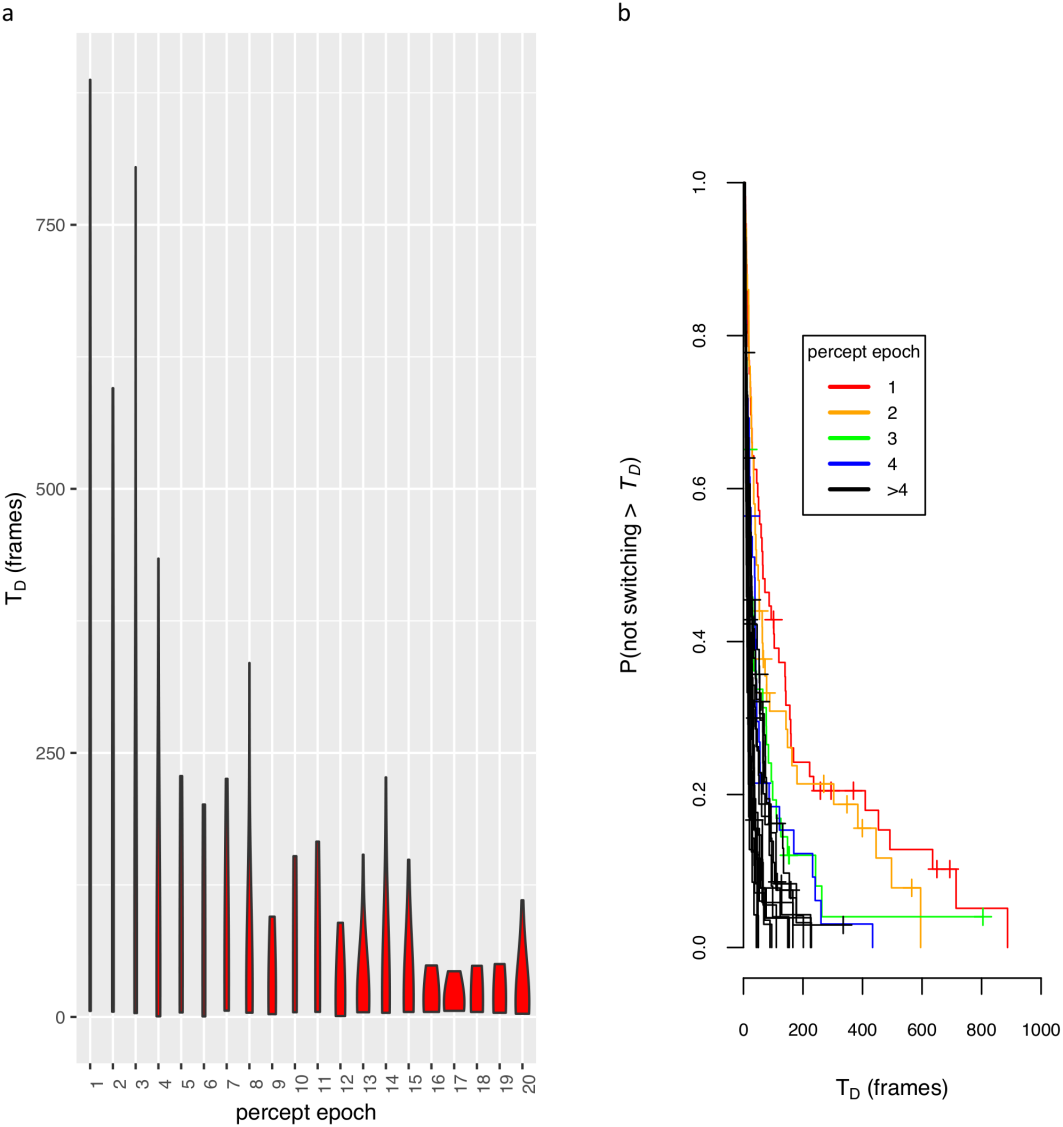
Habituation survival analysis for pooled hypothesis-naive subject data. **(a)** Violin plots for pooled data of T_D_ vs. percept epoch (the order of reported percepts). **(b)** Survival plots for the same analysis for various epochs.

For the hypothesis-naive experiment we discovered a larger habituation effect of the percept durations over percept epochs. The initial percept epochs had longer T_D_ as shown by Fig 5, and we estimated a hazard ratio of 1.024 (s.e. = 0.008) (*P* = 2 × 10^−3^; Wald test using 1,322 samples and 1,266 recorded switch events). We used this habituation effect as a second constraint for the model.

### Model Results

#### Model equations

Given the degrees of freedom and the symmetries in the quartet illusion, as seen in Fig 2a, a minimal model of the quartet illusion involves competition between four types of neuronal pools representing H+, H-, V+ and V- in the presence of fatigue processes. The rate-model dynamics of the canonical cortical circuit for pool *i* are described by:

$$\begin{array}{cc} & \tau_{u}\frac{du_{i}}{dt}=-u_{i}+f_{i}\left(S_{i}+\sum_{j=1}^{N}s_{ij}\beta_{ij}u_{j}-\gamma a_{i}\right)\\ & \tau_{a}\frac{da_{i}}{dt}=-a_{i}+u_{i}\\ & \tau_{s}\frac{ds_{ij}}{dt}=1-s_{ij}-\phi s_{ij}u_{j} \end{array}$$ (1)

where *N* is the number of pools, *f*_*i*_ is a gain function that is zero below a threshold and monotonically increasing above the threshold, *u*_*i*_ is the activity of pool *i*, *S*_*i*_ is the effective input strength to pool *i*, *β*_*ij*_ is the synaptic strength from pool *j* onto *i*, *s*_*ij*_ is a nonlocal fatigue variable that results in an activity dependent decrease of synaptic strength such as synaptic depression [36], *ϕ* governs the nonlocal fatigue strength, *a*_i_ is a local fatigue variable that decreases the activity of the pool through negative feedback such as due to spike-frequency adaptation or synaptic depression of recurrent connections within a pool [12,36,37], and *γ* is the local-fatigue strength. In our ensuing simulations and analysis we assumed that the gain functions are the same for all pools. We also used a gain function that is a square root for positive argument and zero for negative but we probed arbitrary gain functions theoretically. Also, without loss of generality, we can rescale time so that *τ*_*u*_ = 1 and choose the gain threshold to be zero by shifting *f*. We verified that the value of *τ*_*u*_ does not affect our results as long as it is much smaller than *τ*_*a*_ or *τ*_*s*_, as in the static rivalry analysis [38]. For both local and nonlocal fatigue we use time constants on the order of seconds consistent with prior static rivalry papers [11,22]. This falls within the wide physiological range for fatigue analogs such as spike-frequency adaptation or synaptic depression [39–41].

We assume that the relevant input to the pools is a brief stimulus during the transition between frame presentations and that there exists a network that detects change, which is known to exist physiologically (i.e. computes a derivative) [42]. The details of the change detector are not important for the model. Two of the four pools receive an input during a transition from one stimulus parity to the other. For example, a transition from UR-LL to LR-UL provides stimulus to V+ and H- whereas in the following transition from LR-UL back to UR-LL, V- and H+ are stimulated. We implemented intermittent stimuli by periodic square-wave input pulses, and define the *on-state* as the time during the input stimulus pulse and the *off-state* as the time between pulses. Thus *S*_*i*_ alternates between the on-state input *S*_on_ and a background off-state input *S*_off_. The background input S_off_ was modeled as either a fixed low-level input (which can be zero) or a random input given by S_off_~N(0, *σ*^2^). Consistent with neuronal recordings, we presume that the activity imbalance between the pools determines which object dominates perception (i.e. the most active pool corresponds to the percept) [2,3].

For the quartet illusion, we used four pools, each representing one of V+, V-, H+, and H-. If mutual inhibition between pools (negative *β*) is sufficiently strong then only one of the pools is active in an on-state. Given that one of two states is possible at any given on-state, there are 2^n^ possible orbits for a train of *n* on-states (with possible transitions shown in Fig 2b). However empirically, subjects tend to observe only two and occasionally four of these possible orbits; i.e. H or V oscillations, or + or—rotations. This constrained pattern of activation indicates a strong restriction of orbits in the system. The rivalry between orbits suggests the action of a fatigue process. By the symmetries of the quartet model, the persistence of one degree of freedom (direction or orientation) immediately implies oscillations in the other. This can be achieved by biasing the positive connection weights between like-orientations (V+ to V- and H+ to H-) and like-directions (V+ to H+ and V- to H-) as shown in Fig 2a.

In the following, we examine three possible mechanisms in this quartet model for holding the memory required for rivalry between a restricted set of orbits in the presence of biased connections. In the first mechanism, the memory is held by the fatigue variable, in the second, the memory is held by persistent activity due to excitatory connections, and in the third, the memory is held by mutual inhibition driven by nonspecific background activity. We argue that the third mechanism is the most plausible.

#### Fatigue-based mechanism

Persistent V or H orientation illusory motion is automatically obtained if a switch in direction is forced on each frame presentation. This can be achieved by positive lateral connections between the like-direction pools that allow fatigue to suppress activity in the opposite-direction pools. For example, suppose H+ is dominant when both H+ and V- are stimulated and this induces more fatigue in V+ than H-. On the next presentation, H- will dominate over V+. If on the next presentation H+ dominates over V-, then orientation H will persist. Eventually, the fatigue builds in the dominant orientation so that the opposite orientation becomes dominant resulting in alternations (rivalry) between orientations at a slower time scale. Thus there are two fatigue induced alternation processes, one between directions at short times scales and one between orientations at long time scales. Although rivalry between orientations can occur in such a system, there is a trade-off between the short time oscillations and the long time alternations and we were unable to find a set of parameters that could reproduce dynamic L4 and habituation as observed in our experiments. Additionally, the mechanism relies on the symmetries inherent in the quartet illusion and thus the mechanism does not easily generalize to all forms of intermittent rivalry and rivalry memory. For these reasons, we find that the fatigue-based mechanism to be implausible.

#### Persistent activity mechanism

**Fig 6:**
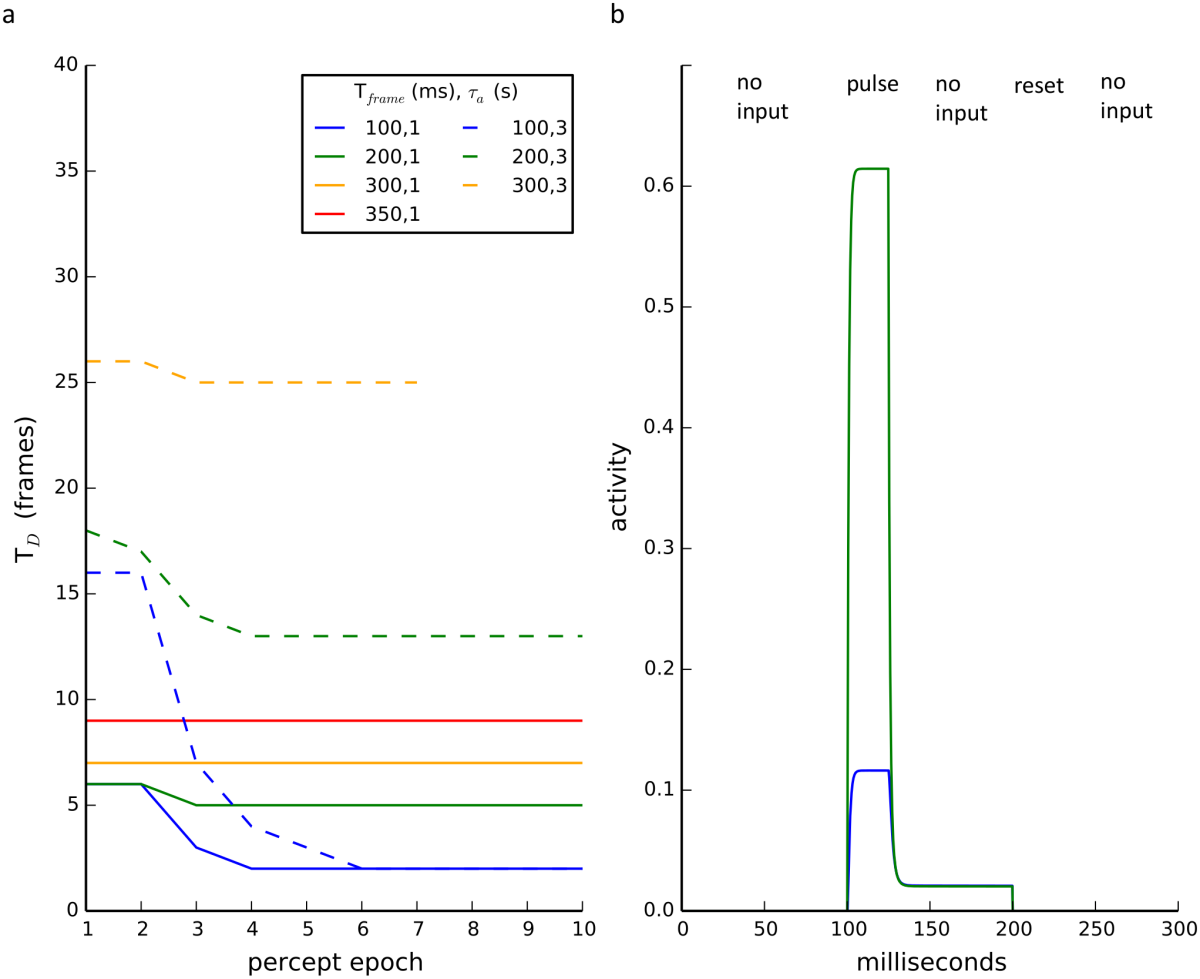
Persistent activity maintains memory. **a)** Dominance durations (T_D_) as a function of percept epochs starting from rest for different T_frame_ intervals and fatigue time constants. A subset of the T_frame_ conditions tested showed habituation. When the fatigue time constant was increased there was both an overall increase in dominance durations and all of the rivalry states showed habituation. See S2 Text for model specifications. **b)** Like-orientated pools are bistable. When dominant (and receiving nominal inhibition from the suppressed pools) one set of like-orientated pools (H or V) can have persistent activity. Here the pools were initialized with zero activity (no input), then one of the two (green) was given a brief input (pulse), followed by no input, and then a hard reset of their activity to zero. The pulse elicited non-zero activity in both pools but was greater for the pool with direct input. Then instead of falling back to zero activity, both pools remained in an elevated state despite a lack of input. When their activity was forced to zero (reset), they then remained inactive.

A possible mechanism to hold a memory is persistent activity induced by recurrent excitation [43]. This type of mechanism has been explored extensively for short-term memory[14,18,27,37]. Like recurrent excitation, strong reciprocal excitation can also induce bistability in our model. If the strength is within a critical window, we observed rivalry memory and intermittent rivalry. Fig 6a shows the simulated percept durations (T_D_) measured in the number of pulses (on-states) as a function of percept epoch, on-state interval (T_frame_), and fatigue time-constant. T_D_ is seen to increase with T_frame_ at a given epoch exhibiting dynamic L4. The decrease in T_D_ as a function of epoch shows that habituation is observed for a number of stimulus conditions. As shown in Fig 6b, like-oriented pools take on one of two activity states (a low or high state).

Although the persistent activity mechanism is able to reproduce the experimental observations for the quartet illusion, the amount of excitation must be restricted to a very narrow range. Too little excitation eliminates the memory and too much eliminates plasticity. Recurrent excitation also reduces or even eliminates the region for static rivalry and can induce self-oscillations (i.e. rhythmogenesis) [29,38]. Persistent activity would thus call for some mechanism to finely tune the excitatory strength and moderate the effects of synaptic plasticity. The third mechanism detailed below explains all the experiments with fewer constraints.

#### Background activity mechanism

**Fig 7:**
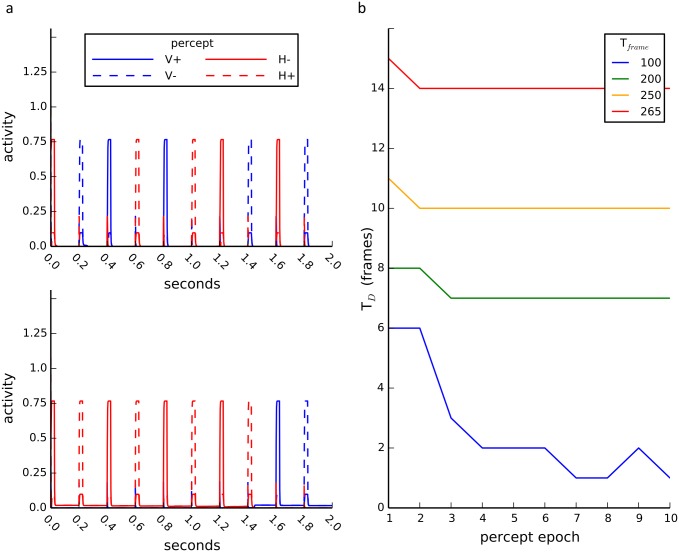
Background-activity stabilizes memory. **a)**(Top) No background activity results in fast switching between orientations for frame interval (T_frame_) of 200 milliseconds. (Bottom) Inclusion of small nonspecific background input results in non-zero activity during off-states (same on-state input as in above). Orientation persists for multiple frame transitions. **b)** Dominance duration (T_D_) increases with T_frame_. All cases show habituation over percept epochs with a larger effect for short intervals. See S2 Text for model specifications.

The third mechanism exploits the fact that low-level background neural activity is the norm experimentally. *In vivo* recordings find that neurons exhibit nonzero resting firing rates in the absence of input and even during suppression [2,7,44–46]. As shown in the top panel of Fig 7a, below the critical persistent activity level positive coupling between like-orientation pools is insufficient to maintain memory of the last orientation. However, in the presence of background activity (see bottom panel of Fig 7a), the previously dominant pool (or reciprocally coupled pools such as like-orientation sensitive pools) in the on-state remains dominant in the off-state even in the presence of fatigue. This dominance is extremely robust to parameter changes, can persist for arbitrarily long off-state durations and represents a true memory state (as we explain in the next section). The model with background activity and local fatigue exhibits both dynamic L4 and habituation (see Fig 7b). Positive coupling in the model biases the circuit towards V/H oscillations rather than rotation but as we show in detail below, it is the background activity together with mutual inhibition that maintains the memory.

#### Reduced two-pool circuit

**Fig 8:**
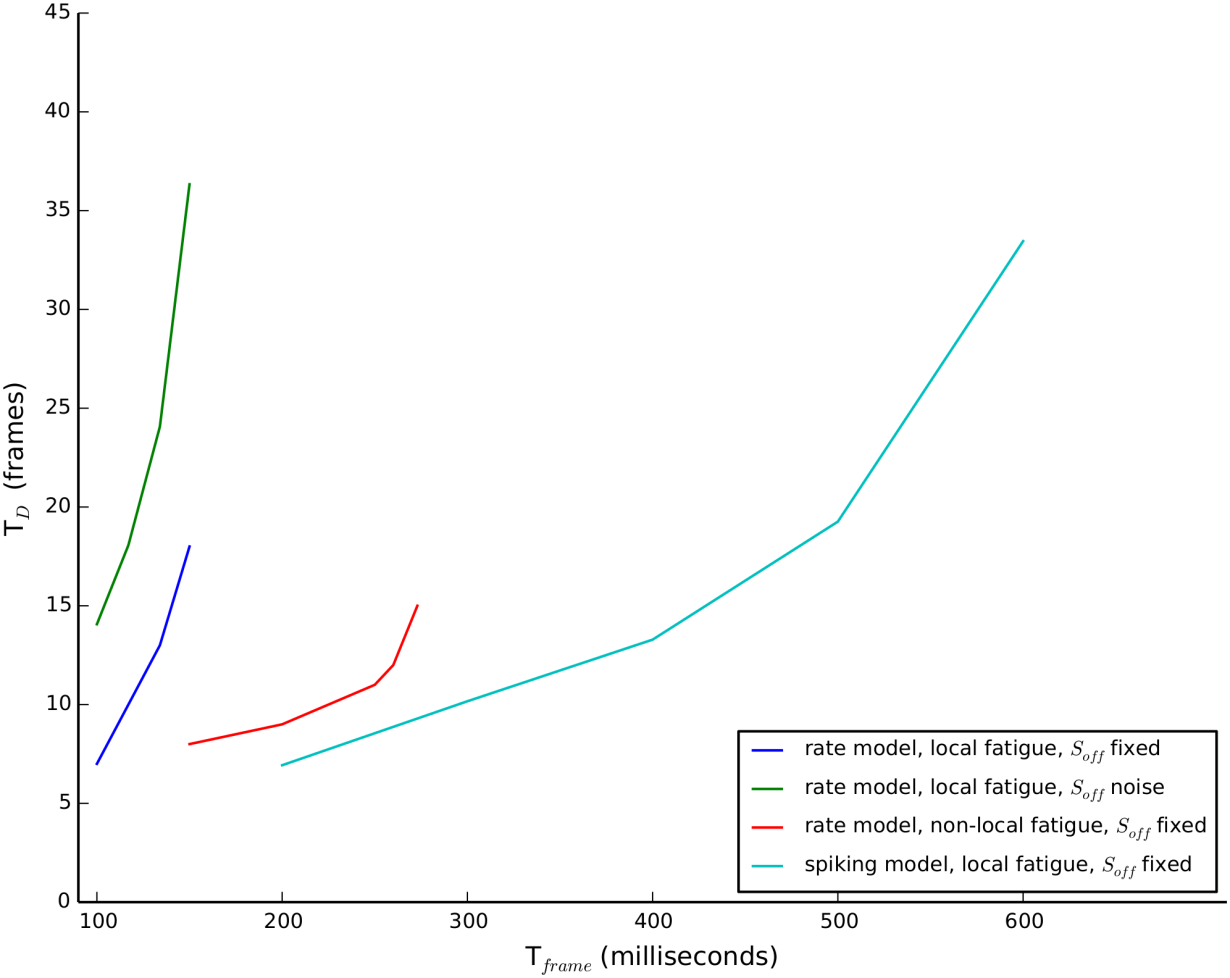
Dynamic L4 for two-pool system under different assumptions. Rate models with either fixed or noisy background input (S_off_) and local or nonlocal fatigue and a conductance-based model with local fatigue all showed dynamic L4. For T_frame_ to the right of a curve we observed no alternations for the duration of the simulation. See S2 Text for model specifications.

**Fig 9:**
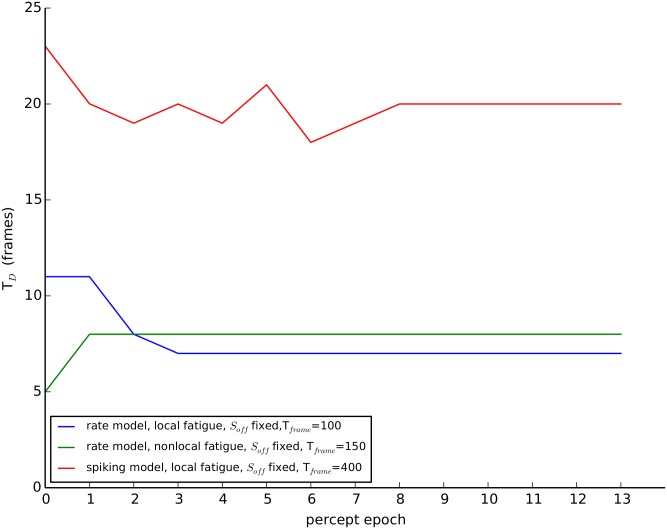
Habituation of percept duration over percept epochs. Simulation results using local-fatigue only or nonlocal fatigue only models with the variables initialized at resting state. For local fatigue there is a decrease in the dominance duration (T_D_) for subsequent dominance periods. For example the first dominance duration (epoch one) is longer than the second. This was seen for both the rate model and the spiking model. For nonlocal fatigue in the rate model the first epoch is shorter than the following epochs. See S2 Text for model specifications.

The background-activity mechanism for memory is best understood in a reduced two-pool model using Eq (1) where N = 2 and we average over the fast directional oscillations, only considering mutual inhibition between orientation (H, V) pools as in Fig 2c. The excitatory connections between like-orientation pools have been subsumed within the pool. They could have been explicitly included as a recurrent (self-to-self) excitatory connection and the persistent activity mechanism would require its presence. However, we show below that the background activity mechanism does not require it. The model with background activity exhibits dynamic L4 for a wide range of parameters (see Fig 8). The background activity could be due to either a constant input, a low threshold of the gain function or even adding zero-mean noise. Zero-mean noise is able to induce a background activity because the nonlinear gain function acts like a rectifier and biases the contribution of the noise to positive fluctuations for durations that are long relative to the time constant of the neural activity. Rivalry memory can also be achieved using a subtractive adaptation-only (local fatigue) model; shunting adaptation is not necessary for rivalry memory as previously proposed [28]. The results held for a system with nonlocal fatigue such as cross-synaptic depression and a more biologically detailed model with conductance-based neurons. We observed habituation for local fatigue but the opposite for nonlocal fatigue (see Fig 9), which we explain below. While the dynamic L4 effect was very robust to model conditions and noise, the habituation effect was somewhat less robust. Habituation was not found in the parameter regimes we searched when background activity was induced by zero-mean noise alone in the rate model. The parameter regime that exhibits both dynamic L4 and habituation was also smaller in the conductance-based model than the rate model, probably because we did not have enough neurons in our spiking model. Our conductance-based model only used a small number of neurons for computational expediency and thus is subject to strong finite-size noise effects.

#### Explanation of background-activity memory mechanism

**Fig 10:**
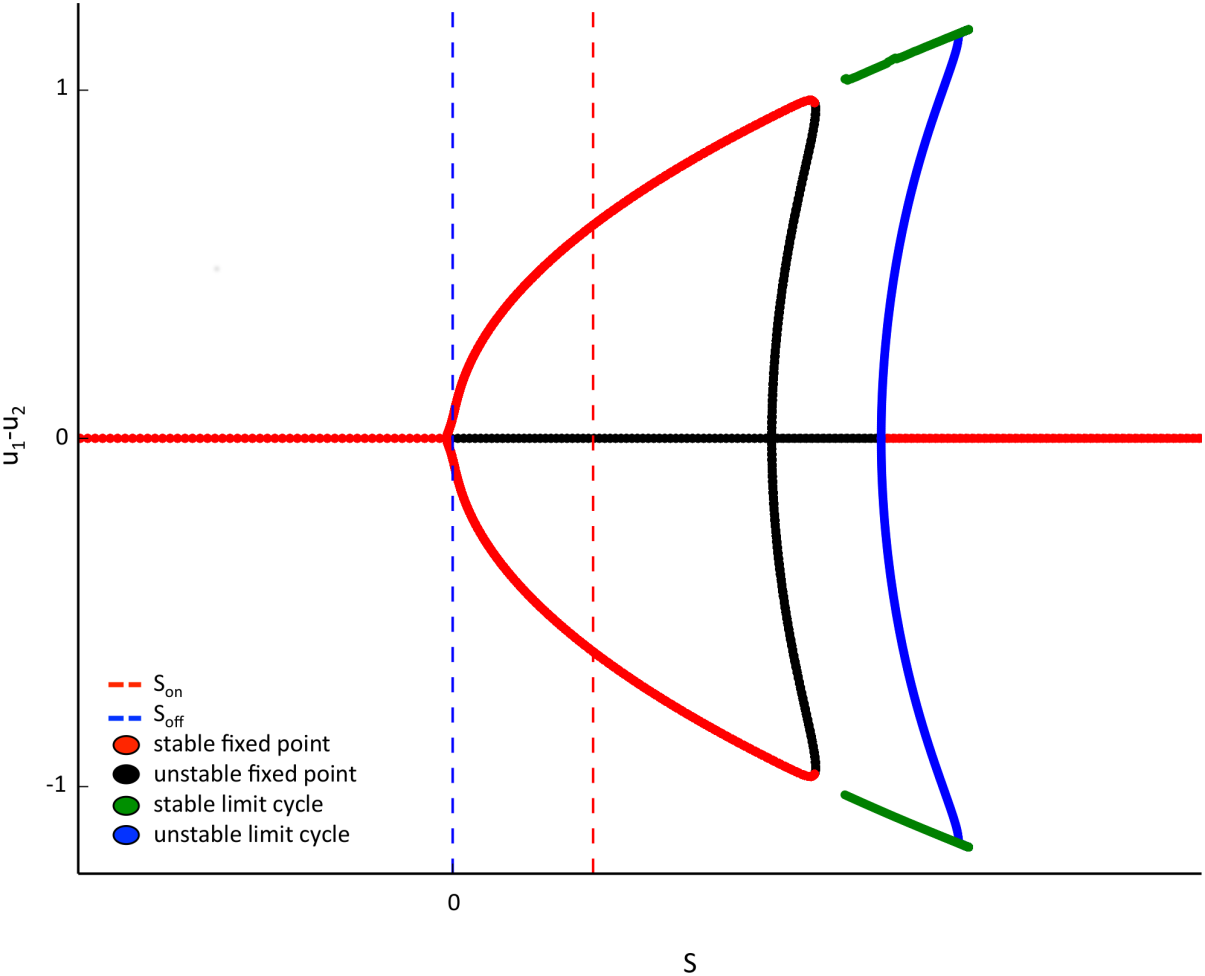
u1-u2 bifurcations across static symmetric external drive S for system with nonlocal fatigue. The pitchfork bifurcation for arbitrarily low input provides the asymmetry necessary for intermittent rivalry and rivalry memory. It allows for memory of the last dominant state by maintaining asymmetry for very small (background) input (S_off_) (S_off_ = 0.001). A shift in the asymmetric state in the off-state by a build-up of fatigue due to larger S in the on-state (S_on_) eventually leads to a shift in dominance. Although not drawn, the limit cycle extends to the red pitchfork terminating in a SNIC. A smoothed threshold was used to generate the plot. For actual simulations we used a hard threshold and zero input gives symmetric, zero activity.

The bifurcation plot of *u*_1_-*u*_2_ versus a static *S* for the reduced two-pool circuit model is shown in Fig 10. The bifurcation structure for static input is pertinent when the model is applied to intermittent rivalry and the quartet illusion as seen next. For negative *S* there is a symmetric phase (*u*_1_ = *u*_2_). As *S* is increased there is a pitchfork bifurcation to an asymmetric phase (winner-take-all state) at *S* = 0, where the sign indicates the dominant pool. For larger *S* there is a saddle node on a limit cycle bifurcation (SNIC) to static rivalry. Depending on the parameters, including the shape of the gain function, the static rivalry state can cease on a supercritical Hopf bifurcation or, as seen in Fig 10, on a SNIC with a region of bistability between rivalry and a symmetric state, which ends on a subcritical Hopf bifurcation. These phases can be stretched, shifted, or not exist. Additional features can also appear such as another Hopf bifurcation or period doubling bifurcations depending on the parameters (see [22,38] for more detailed review). As we will show, the presence of the asymmetric state at very low levels of input is the crucial feature for intermittent rivalry and rivalry memory.

First, consider the case where the two pools have no background activity in the off-state as in the standard static rivalry model. This corresponds to below threshold input in Fig 10 (the activities need not be zero but can be any symmetric state where *u*_1_ − *u*_2_ = 0 is the fixed point). For this case there is a switch in the dominant percept with each pulse because each pulse weakens the dominant pool and this persists until the next pulse. Thus there is an initial bias favoring the last suppressed pool. Under these conditions, the standard rivalry model would not explain rivalry memory or intermittent rivalry, agreeing with previous assertions that this model is insufficient for rivalry memory and must be augmented [28,47,48].

However, including background activity during the off-state (as in the four pool model above) produces rivalry memory since it maintains the activity asymmetry at an arbitrarily low activity level. This can be appreciated in the bifurcation diagram by the existence of the asymmetric state close to zero (see Fig 10). There are two competing biases in the system. One is held by the fatigue variable and the other is held by the low-activity asymmetric state due to mutual inhibition and low-level background activity. Initially, the low-activity memory will dominate and thus the previously dominant state will remain dominant. However, the fatigue variable will become stronger for each subsequent on-state and thus after a sufficient number of on-states, it will eventually overcome the low-level activity bias and the dominance will switch. The model also preserves all of its original properties like static rivalry and normalization, since the low level activity does not play a role during the on-state.

We can make these observations more mathematically precise. A requirement for mutual-inhibition memory is that the asymmetric state must exist at low levels of input strength. This is easily achieved if the inhibition is strong enough and the gain function is concave like a square root function. The gain function need not be concave everywhere but just over some range. From Eq (1), the fixed point is given by

$$u_{1}=f\left(S-s_{2}\beta u_{2}-a_{1}\right)$$

$$u_{2}=f\left(S-s_{1}\beta u_{1}-a_{2}\right)$$

where we set s_ij_ to s_j_ for notational simplicity. Consider the asymmetric state (one pool is dominant) where *u*_1_ > 0 and *u*_2_ = 0. This requires

$$s_{1}\beta f\left(S-a_{1}\right)+a_{2}>S>a_{1}$$ (2)

First consider the case of no fatigue (i.e. set *s* = 1 and *a* = 0) in which case Eq (2) becomes *βf*(*S*) > *S* > 0. If *f* is a concave function and *f*(*x*) = 0 (i.e. *f* (*tx*) ≥ *tf* (*x*),0 ≤ *t* ≤ 1) then if this condition is satisfied for *S*_on_ then it will also be satisfied for any 0 < S_off_ ≤ S_on_. As shown in the S1 Text, there is still a wide range of S_off_ that satisfies Eq (2) in the presence of fatigue. Thus the asymmetry in the on-state can be held in the off-state by the background activity. The memory is topological because the state is a discrete invariant for a wide range of parameter values.

The percept dominance will switch when condition Eq (2) no longer holds. During the off-states the fatigue variables tend to relax to a background but during the on-state *a*_1_ will increase and *s*_1_ will decrease. Thus a dominance switch can occur through a decrease in *s*_1_*βf* (*S* − *a*_1_) + *a*_2_ below *S*_on_ or an increase in *a*_1_ above *S*_off_. The former mechanism has been called *escape* since the amount of inhibition becomes too small to suppress the inactive pool and the latter *release* since the dominant pool can no longer remain active [22,38]. In both mechanisms an increased frequency of on-state presentations will lead to a faster increase in the fatigue variable and hence a shorter dominance time. This is in contrast to static rivalry where increasing input strength shortens dominance time for escape but lengthens it for release. Also note while static rivalry can only occur if fatigue and input is sufficiently strong to produce a limit cycle, this is unnecessary for intermittent rivalry. Intermittent rivalry can occur for either an asymmetric on-state or a limit cycle as long as there is asymmetry in the off-state. If the on-state is a limit cycle, then for pulses longer than the limit cycle period we expect to see mixed static and intermittent rivalry results with alternations during the on-state. This is consistent with prior experiments [32]. The dominance time will also have a strong nonlinear dependence on *T*_frame_ when the maximal fatigue is near threshold. We give detailed proofs in the S1 Text.

For the rested system, local fatigue is sufficient to reproduce habituation, but nonlocal fatigue gives the opposite results. We tested if the model exhibited the slow habituation we observed in the experiment without any fine-tuning of the parameters (see Fig 5) in the local and nonlocal fatigue models for the fully rested system (local fatigue variables initialized to zero or nonlocal fatigue to one). As shown in Fig 9, only local fatigue produces habituation over several epochs. For nonlocal fatigue the first epoch is generally shorter than the rest although for some initial conditions the first epoch can be longer. We show below that the difference between local and nonlocal fatigue is due to how the two fatigue variables induce dominance alternations.

**Fig 11:**
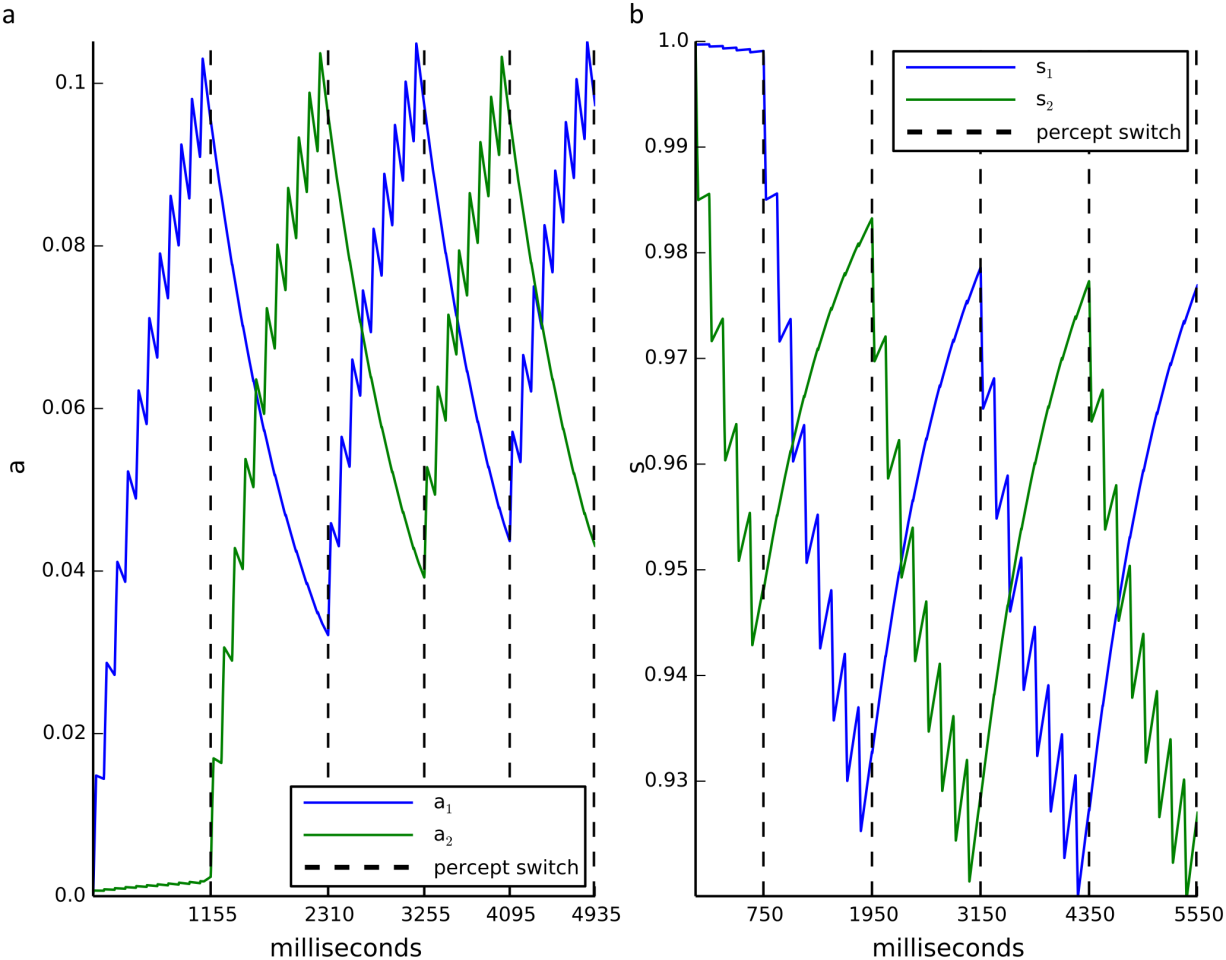
Local versus nonlocal fatigue variables for the rested rate-model. **(a)** Local fatigue variable a initialized to zero for pools i and j over percept epochs (T_frame_ = 105 milliseconds). **(b)** Nonlocal fatigue variable s initialized to one for pools i and j over percept epochs (T_frame_ = 150 milliseconds). See S2 Text for other model specifications.

Fig 11 shows the simulated fatigue variables of the two populations *i* and *j* across percept epochs. Note that the fatigue orientations are reversed in Fig 11 since local-fatigue increases with increased input; whereas, the nonlocal fatigue variable (cross-pool synaptic strength) decreases with increased input. Recall that a switch is triggered either due to the dominant population falling below the threshold (release) or the suppressed population rising above threshold (escape). For local fatigue, the threshold required to erase the low level off-state activity is fixed (as seen in Fig 11, the variable increases to approximately the same value for each switch). The dominance time is given by the time it takes the local fatigue variable to reach the fixed release threshold and this time depends on its starting value, which is low for the first epoch when the system is well rested. Thus, the dominance time will scale linearly with the fatigue decay time (*τ*_*a*_ or *τ*_*s*_) and logarithmically with *S*_off_ (where log is negative and can vary quickly). The suppressed pool fatigue does not relax back to its previous starting value if its rate of recovery is slower than the rivalry rate. Hence, the dominance time for subsequent epochs will shorten until a steady state is reached. The gradual decrease in dominance time matches the experimentally observed habituation.

Nonlocal fatigue behaves differently. It cannot erase the dominant pool off-state activity; instead, it works by decreasing the inhibition on the repressed population until it can escape. A switch occurs when the ratio of the inhibition on the dominant pool to that on the suppressed pool drops below a critical value. The dominance time is given by the time it takes this ratio to reach the critical value starting from some initial condition. In the completely rested state, fatigue of the first dominant population has a shorter distance to traverse to reach the critical ratio than the dominant pool in the second epoch (see Fig 11b). Hence, after the first epoch the dominance time is relatively constant because any global accumulation of depression on both synapses effectively cancel out, which is not in agreement with the experimental observation (see S1 Text for details). The initial ratio could be manipulated such that the first epoch is longer but this still does not match the data. In summary, the mathematical analysis explains the conditions for intermittent rivalry and suggests that local fatigue, for example by spike frequency adaptation or local synaptic depression, is the dominant form of fatigue for intermittent rivalry that exhibits gradual habituation like the quartet illusion.

## Discussion

We examined several intermittent rivalry and rivalry memory mechanisms in a canonical cortical circuit model and concluded that the inclusion of experimentally observed low-level, nonspecific background neuronal activity that induces a mutual-inhibition memory is the most plausible. The mechanism preserves all the phenomena of static rivalry as shown in previous studies [11,12,22]. Mutual inhibition has been proposed before as a mechanism for short-term memory [27]. However, it was not considered for rivalry since it was assumed that the circuit could not maintain the memory in the absence of a stimulus and the presence of fatigue would favor the suppressed percept. Even if neurons are not completely inactive during the off-states, it is not *a priori* obvious that an asymmetric state would exist and persist indefinitely in the presence of fatigue. We show that for reasonable conditions, this form of memory is topological (holds for a wide range of parameters) and stable for arbitrarily low input. Topological mutual-inhibition memory can exist as long as inhibition is strong enough and the gain function (i.e., frequency-input curve) has a non-increasing slope, which is experimentally observed [36,49]. Thus, it may not be limited to rivalry and could be the mechanism for other forms of memory in the brain.

The model explains the two intermittent rivalry phenomena of dynamic L4 and habituation in the quartet illusion. Dynamic L4 is similar to what Leopold et al. (2002) found for the rotating sphere (RS) stimulus using manually inserted blank periods. A habituation effect similar to what we found for the quartet has also been noted for other stimuli (e.g., static images, motion) and sensory domains (e.g., visual, auditory) [33–35]. However, binocular rivalry shows the opposite effect where the switch rate decreases over epochs. This has been attributed to contrast adaptation [50] and can be accounted for in the rivalry model by reducing input over time. The dynamics of our model are geometrically similar to the model proposed by Noest et al. 2007[28]. However, instead of requiring a subthreshold current with shunting adaptation, we show that the activity itself acts as the memory with the property of persisting indefinitely. There are many possible mechanisms for the background activity and perhaps the most interesting is that zero-mean noise is sufficient. While either local (e.g., spike-frequency adaptation, recurrent synaptic depression) or nonlocal (e.g., cross-pool synaptic depression) is sufficient to explain intermittent rivalry, local fatigue in particular reproduces habituation.

Prior experimental designs of intermittent rivalry introduced the off-states by either manually removing a static stimulus or by utilizing a more complex protocol such as combining motion-induced blindness with a static stimulus, or by instructing the subject to independently attend to mixed “static, intermittent” epochs[10,32]. It is possible that some of these more complex paradigms involve higher order processing that makes replication difficult. We propose that the quartet is a robust self-contained system for the study of intermittent rivalry since periodicity in the quartet invokes natural motion processing. In retrospect, our experimental design was suboptimal for detecting dynamic L4, but the model provides guidance for future experiments. Further study is necessary to determine whether different fatigue modes are important for different rivalry stimuli. This could be predicted with the model and tested experimentally by behavioral and electrophysiological measurements, perhaps combined with pharmacological manipulation of K^+^-channels that modulate adaptation effects [51]. Finally, the role of background activity in the theory predicts that perturbing or suppressing the activity bias will extinguish rivalry memory.

## Methods

### Ethics Statement

Ethics approval for this study was granted by the NIH Combined Neuroscience Institutional Review Board under protocol number 10-M-0027 (ClinicalTrials.gov ID NCT01031407).

### Subjects

The naive-subject study consisted of 16 adult male participants from the National Institutes of Health campus and DC metropolitan region. The data were screened such that at least two percept switches were reported in a test block. One subject did not have adequate data and was removed, leaving 15 subjects. Author data was collected on SV and PT for the quartet.

### Task Design

Naive-subjects were tested on the standard quartet task. The standard quartet animation consists of two alternating still frames where a single frame consists of one set of dots located at opposing corners of an upright square as shown in Fig 1. The task was administered in a dark room with maximum screen and keyboard brightness (approximately 2 lux) on a 15 inch 2010 MacBook Pro with 60 Hz frame rate. Our stimulus consisted of three nested quartets centered on the viewing screen as shown in Fig 1 (also see S1 Movie); chosen to mitigate the Troxler effect and to induce motion perception over a large receptive field. The dots were outlined with the RGB color = 46, 55, 254 and set on a black background. For a typical viewing distance of 24 inches, although this was not rigidly enforced, the square length visual angle (dot diameter visual angle) of each nested quartet were 5.5 (1), 3.5 (0.5), 1.5 (0.25) degrees. There was a fixed orange, central dot with RGB color = 255,127,0. This was the instructed focal point for the subject. These parameters were arbitrarily chosen. During the task, subjects were instructed to maintain fixation on this central dot and report a change in motion by pressing the Return-key. To give feedback that a switch was recorded, the central dot briefly changed color from orange to green (RGB = 0,255,0) to orange when the subject reported a change.

The task consisted of 7 blocks: a tutorial, a task-comprehension block, and 5 frame-period (T_frame_) blocks with a two second black screen period between blocks. Participants were told that the task would test their ability to detect changes in motion and were not informed that the motion was ambiguous. They were also shown examples of the types of motions to expect: horizontal or vertical, consisting of unbiased apparent motion animations, where a set of three frames was repeatedly presented such that the dots progressed from corner, to center, to corner, to center. This was done for vertical and horizontal orientations of motion. The task-comprehension block used this same biased motion stimulus and the direction was changed at fixed intervals that were known to the experimenter. The first frame-period block was set to 300 millisecond frame intervals and lasted six minutes (we refer to this as the practice block). This was followed by four blocks with frame periods (and block durations) of 300 (5), 350 (5), 400 (6), and 450 (8) ms (minutes). The order of the last four blocks was shuffled between participants.

Author-data was collected on the quartet task. The quartet task was similar to the above but without the tutorial, task-comprehension, or practice blocks. These sessions consisted of randomized T_frame_ with 2–3 sampled frame periods and the duration was based on reporting 11 switches (some T_frame_ were repeated in separate sessions).

### Statistical Analysis

Data from the naive-subject group was analyzed using a Cox proportional hazards model (see [52,53] for review). It is a semi-parametric model that is robust to the distribution of the data, which was non-normal for our case. The analysis calculates statistics across all time points and accounts for right-censored data. We chose this approach since it accounts for percept duration that may extend beyond the test block, including those that may span the majority of a block. The frequency of percepts that remained dominant after T_D_ dotted frame presentations were fit to a baseline survival model for each test condition (e.g., T_frame_) and normalized (the non-parametric component of the model). The hazard ratio is the exponential adjustment to the baseline survival for the conditioned effects, which is the parametric component. An increased hazard ratio means there was an increased probability of percept extinction over a time period as the parameter was increased. To be considered a valid model fit, there were two statistical tests: 1) test whether the estimated coefficient for the effect is non-zero, and 2) that the distribution of the residuals has zero slope across time (is flat).

We used the *coxme* mixed effects R function to estimate the hazard ratios. The mixed effects model was used so we could account for subject- or T_frame_- specific deviations. A Wald test was used to estimate a *P*-value for the hazard ratio. The *coxme* library does not have a test for the residuals. To check this we calculated a noise vector (***z***) from the *coxme* fit given by ***z*** = ***ŷ*** − *θ****x***, where ***ŷ*** is the linear model prediction, *θ* is the estimated coefficient, and ***x*** is the predictor vector. The noise vector ***z*** was used as an offset in the R function *coxph* and the residuals from this were checked with *cox*.*zph*. We used a criterion of no significant deviation of the residual slope from zero (i.e., *P*>0.05), in order for the proportionality assumption to apply.

### Numerical Analysis

Bifurcation analysis was performed in XPPAUT [54]. Rate models were simulated using Python. Conductance-based model simulations were performed with circuit of two mutually inhibiting neuron pools with a calcium activated spike frequency adaptation-like current and synaptic depression. This model was simulated in XPPAUT and the output was analyzed in Python. All code used for simulations and numerical analysis including parameters are attached as S2 Text.

## Supporting Information

S1 Text Mathematic analysis. (PDF)

S2 Text Numerical analysis. (ZIP)

S3 Text Data. (ZIP)

S1 Movie Stimulus example. (ZIP)
