# Supplementary material for: Canonical Cortical Circuit Model Explains Rivalry, Intermittent Rivalry, and Rivalry Memory
Source: PLoS Comput Biol. 2016 May 3;12(5):e1004903. doi: 10.1371/journal.pcbi.1004903 (PMC4854419; doi:10.1371/journal.pcbi.1004903)
Supplement: S1 Text — (PDF) [file pcbi.1004903.s001.pdf]

Supplementary Information: Mathematical Analysis

We establish some mathematical conditions for when 1) mutual-inhibition induced topological memory, 2) intermittent rivalry, 3) dominance time dependence on frame presentation period, and 4) slow habituation effect will arise in the rivalry model for $i = 1, 2$:

$$\tau_u \frac{du_i}{dt} = -u_i + f(S(t) - \beta s_j u_j - \gamma a_i) \tag{1}$$

$$\tau_a \frac{da_i}{dt} = -a_i + u_i \tag{2}$$

$$\tau_s \frac{ds_i}{dt} = 1 - s_i - \phi u_i s_i \tag{3}$$

where $f(x) = 0$ for $x \leq 0$ and it is a monotonically increasing continuous function for $x > 0$. $f(x)$ is differentiable everywhere except possibly at $x = 0$. Note that this is still general because we can always shift the threshold to zero by shifting x. $S(t)$ oscillates between an an off-state $S_{\text{off}} > 0$ for time T_{off} and an on-state $S_{\text{on}} > S_{\text{off}}$ for time T_{on}, with a period of $T = T_{\text{on}} + T_{\text{off}}$. The initial conditions satisfy $0 < u_i(0) < f(S_{\text{on}})$, $0 \leq a_i(0) < f(S_{\text{on}})$, and $0 < s(0) \leq 1$.

Topological memory requires that there exists a winner-take-all (WTA) fixed point where one population is active while the other is silent in both the off-state and the on-state. The fixed point obeys

$$u_i^0 = f(S_{\text{off/on}} - \beta s_j u_j^0 - \gamma a_i) \tag{4}$$

for fixed a_i and s_j. The WTA fixed point consists of a state where the activity of one pool is zero while the other is nonzero. Without loss of generality, let us choose $u_1^0 > 0$ and $u_2^0 = 0$. Topological memory implies the existence of a WTA fixed point for a wide range of inputs satisfying the conditions:

$$u_1^0 = f(S - \gamma a_1) \tag{5}$$

$$u_2^0 = 0 \tag{6}$$

The existence condition requires $S > \gamma a_1$, $S - \beta s_1 u_1^0 - \gamma a_2 < 0$. Hence, topological memory is possible if

$$\beta s_1 u_1^0 + \gamma a_2 > S > \gamma a_1 \tag{7}$$

Theorem 1. *In the absence of fatigue ($s_i = 1$ and $a_i = 0$), for S on any finite interval $\Omega = [r_1, r_2]$, $r_2 > r_1 > 0$, there is a $\beta > 0$ such that topological memory exists.*

Proof. Without fatigue, the existence condition for topological memory is $\beta f(S) > S > 0$. Hence, S must be positive by the lower bound condition. Since $f(S)$ is positive and continuous by definition for $S > 0$, $S/f(S)$ is bounded and continuous and the upper bound condition can be written as $\beta > S/f(S)$. Now pick an interval Ω. Since $S/f(S)$ is continuous and bounded there is a supremum C on the interval. Taking $\beta > C$ satisfies $\beta > S/f(S)$ as required. $\square$

Theorem 2. *If the fixed point is WTA for $S = S_{\text{on}}$ then it is also WTA for $S = S_{\text{off}} < S_{\text{on}}$ if f is concave on $S > 0$ (e.g. $f'' < 0$).*

Proof. The WTA fixed point for $S(t) = S_{\text{on}}$ satisfies $\beta f(S_{\text{on}}) > S_{\text{on}} > 0$. Hence, $\beta > S_{\text{on}}/f(S_{\text{on}})$ since $f(S_{\text{on}}) > 0$. We must show that this implies $\beta f(S_{\text{off}}) > S_{\text{off}} > 0$. The lower bound is satisfied by the definition of S_{off}. The upper bound requires $\beta > S_{\text{off}}/f(S_{\text{off}})$. Because f is concave then $f(tS) \geq (1-t)f(0)+tf(S) = tf(S)$, $t \in [0, 1]$, since $f(0) = 0$ by definition. Let $S_{\text{off}} = tS_{\text{on}}$. Then $S_{\text{off}}/f(S_{\text{off}}) = tS_{\text{on}}/f(tS_{\text{on}}) \leq S_{\text{on}}/f(S_{\text{on}}) < \beta$ by concavity, which proves the proposition. $\square$

Corollary 1. *Topological memory can persist with nonzero fatigue ($a > 0, s < 1$) for some choice of S_{off}, S_{on}, and $\beta > 0$.*

Proof. Topological memory requires that (7) must be satisfied. Choose $S_{\text{off}} > \gamma a_1(t)$ so that lower bound condition is satisfied. Since f is a continuous positive function for positive argument and $S \geq S_{\text{off}}$ is positive and bounded then $u_1(t)$ is positive and bounded. Hence, $a_i(t)$ is positive and bounded and $0 < s_j \leq 1$. u_1^0 from (5) is also positive. Hence, for any γ, (7) can be satisfied for sufficiently large β. $\square$

Remark 1. *The piecewise square root gain function $f(x) = \sqrt{\max(x, 0)}$ satisfies all the conditions for topological memory.*

We have thus established the conditions for topological memory. We must now examine the dynamics to probe the existence of intermittent rivalry. We consider the singular limit where the fatigue variables are much slower than the activity variables, i.e. $\tau_a, \tau_s >> \tau_u$, and use a slow-fast time-scale

analysis. Without loss of generality, we rescale time such that $\tau_u = 1$. The activity variables are a fast subsystem while the fatigue variables comprise the slow subsystem. In the singular limit, we can examine the fast dynamics assuming that the slow variables are fixed.

At the initiation of the on-state, $S(t)$ changes abruptly from S_{off} to S_{on} at which point the off-state fixed point no longer exists. The system will then evolve towards the asymmetric on-state fixed point. The pool that becomes dominant depends on which basin of attraction the off-state fixed point resides, i.e. which side of the separatrix the off-state lies on. We can compute the direction of the initial response to the on-state by linearizing around the off-state fixed point with $u_i(t) = u_i^0 + v_i(t)$. Using (4), the fast subsystem then obeys

$$\frac{dv_i}{dt} = A_i - v_i - f_i'(S_{\text{on}})\beta s_j v_j \tag{8}$$

where $f_i(I) = f(I - s_j \beta u_j^0 - \gamma a_i)$, and $A_i = f_i(S_{\text{on}}) - f_i(S_{\text{off}})$. Setting $\tilde{\beta} = f_i'(S_{\text{on}})\beta s_j$ in (8) gives

$$\frac{dv_i}{dt} = A_i - v_i - \tilde{\beta}_j v_j \tag{9}$$

with initial conditions $v_i(0) = 0$. In matrix form, (9) is

$$\frac{dv}{dt} = A + Mv \tag{10}$$

where $v = (v_1, v_2)'$, $S = (A_1, A_2)'$ and

$$M = \begin{pmatrix} -1 & -\tilde{\beta}_2 \\ -\tilde{\beta}_1 & -1 \end{pmatrix} \tag{11}$$

The eigenvalues of M are $\lambda_\pm = -1 \pm \sqrt{\tilde{\beta}_1 \tilde{\beta}_2}$ with eigenvectors

$$e_\pm = \left(1, \mp\sqrt{\tilde{\beta}_1/\tilde{\beta}_2}\right)' \tag{12}$$

respectively. We can diagonalize the system by operating on (10) with the inverse of the matrix of eigenvectors $U = [e_+, e_-]$:

$$U^{-1} = \frac{1}{2}\begin{pmatrix} 1 & -\sqrt{\tilde{\beta}_2/\tilde{\beta}_1} \\ 1 & \sqrt{\tilde{\beta}_2/\tilde{\beta}_1} \end{pmatrix} \tag{13}$$

to obtain

$$\frac{dv_\pm}{dt} = A_\pm + \lambda_\pm v_\pm \tag{14}$$

where

$$\begin{pmatrix} v_+ \\ v_- \end{pmatrix} = U^{-1} \begin{pmatrix} v_1 \\ v_2 \end{pmatrix}, \begin{pmatrix} A_+ \\ A_- \end{pmatrix} = U^{-1} \begin{pmatrix} A_1 \\ A_2 \end{pmatrix} \tag{15}$$

and initial conditions are $v_\pm(0) = 0$. The solutions are

$$v_\pm(t) = \frac{A_\pm}{\lambda_\pm}(e^{\lambda_\pm t} - 1) \tag{16}$$

If $\sqrt{\tilde{\beta}_1 \tilde{\beta}_2} > 1$ then $\lambda_+ > 1$ and $v_+(t)$ will tend to either positive or negative infinity depending on the sign of A_+, while $v_-(t)$ will approach zero. Hence, asymptotically for large t, we have

$$v_1(t) = v_+ + v_- \sim \frac{A_+}{\lambda_+} e^{\lambda_+ t} \tag{17}$$

$$v_2(t) = \sqrt{\frac{\tilde{\beta}_1}{\tilde{\beta}_2}}(v_- - v_+) \sim -\sqrt{\frac{\tilde{\beta}_1}{\tilde{\beta}_2}} \frac{A_+}{\lambda_+} e^{\lambda_+ t} \tag{18}$$

If $A_+ > 0$ then population 1 will become dominant and if $A_+ < 0$ then population 2 will be dominant. $A_+ = \left(A_1 - \sqrt{\tilde{\beta}_2/\tilde{\beta}_1} A_2 \right)/2$ is a function of the slow variables.

In order to prove the existence of intermittent rivalry, we need to examine how repeated on-states affect A_+. Let T_n represent the time of the nth on-state. $A_+(T_n)$ changes on each on-state because of the change in the slow variables (i.e. fatigue processes). What we need to show is that the change of the slow variables is sufficient to cause A_+ to alternate signs.

Consider first that the only slow process is local fatigue. We can then set

$s_1 = s_2 = 1$. Let population 1 be dominant. Hence,

$$\begin{aligned}
2A_+(T_n) &= f(S_{\text{on}} - \gamma a_1(T_n)) - \sqrt{\frac{f_1'(S_{\text{on}})}{f_2'(S_{\text{on}})}} f(S_{\text{on}} - \beta u_1^0 - \gamma a_2(T_n)) \\
&\quad - f(S_{\text{off}} - \gamma a_1(T_n)) \\
&\equiv f_1(S_{\text{on}}) - \sqrt{\frac{f_1'(S_{\text{on}})}{f_2'(S_{\text{on}})}} f_2(S_{\text{on}}) - f_1(S_{\text{off}}) \qquad (19) \\
u_1^0(T_n) &= f_1(S_{\text{off}}) \qquad (20)
\end{aligned}$$

The following is essentially a restatement of Proposition 1 and 2:

Corollary 2. *There will be a WTA state where one pool is dominant (i.e. $A_+ > 0$ in (19)) if $f'' < 0$ on $S > 0$ (concave) for some $\tilde{\beta} > 0$.*

Proof. We first note that f is a monotonically increasing function. Hence, $f_1(S_{\text{on}}) > f_1(S_{\text{off}})$ and $f_2(S_{\text{on}}) < f_1(S_{\text{on}})$. If $f_i''(S_{\text{on}}) < 0$ (i.e. f is concave) then $f_2'(S_{\text{on}}) > f_1'(S_{\text{on}})$. Since $u_1^0 > 0$, $f_2(S_{\text{on}})$ decreases as β increases and since $f(0) = 0$ then $f_2(S_{\text{on}}) = 0$ for $\beta > 0$ sufficiently large. Hence, by continuity of f, $A_+ > 0$ for some $\beta > 0$. □

If $f_i(S_{\text{on}})$ is not concave then it is possible that $f_2'(S_{\text{on}}) < f_1'(S_{\text{on}})$ and thus depending on the precise shape of f, dominance may or may not be possible. Herein, we assume that $f(x)$ is concave for $x > 0$. Thus even for a state with no asymmetry in the fatigue, any arbitrarily small initial asymmetry or noise will allow one population to be dominant while the other is suppressed. Dominance can also occur in the presence of local fatigue provided that a_2 is sufficiently large and a_1 is sufficiently small.

Intermittent rivalry will occur if A_+ changes sign. Note that immediately after A_+ reaches 0, there is a discontinuity in A_+ since $u_1^0 = 0$ and $u_2^0 > 0$. Thus, as long as A_+ reaches zero and induces a switch, then the new dominance will be established and A_+ will jump to a larger magnitude value. We thus need to prove that there is a strengh of fatigue for which A_+ will change signs and fatigue can attain that value. This is established in the following two lemmas.

Lemma 1. *If $A_+ > 0$ initially, then A_+ will become negative if $a_1 \geq a_{\text{switch}} \equiv S_{\text{off}}/\gamma$. i.e. A dominance switch will take place if the local fatigue amplitude becomes large enough.*

Proof. We analyze how $A_+(T_n)$ changes as a function a_i. Because $u_2^0 = 0$, we can consider the effects of a_1 and a_2 independently. First consider a_1. Suppose that a_1 is sufficiently small such that $A_+ > 0$. An increase of a_1 causes $f_1(S_{\text{on}})$ to decrease, $f_1(S_{\text{off}})$ to decrease, and $f_1'(S_{\text{on}})$ to increase by concavity of f. It also induces $f_2(S_{\text{on}})$ to increase and $f_2'(S_{\text{on}})$ to decrease through the decrease in u_1^0. Thus, the first two terms of (19) combine to decrease A_+ but the third term increases A_+. For a concave function, $f_1(S_{\text{on}}) - f_1(S_{\text{off}})$ increases with increasing a_1 for $f_1(S_{\text{off}}) > 0$. Thus, a switch is guaranteed when a_1 increases to the point that $f_1(S_{\text{off}}) = 0$, which occurs when $S_{\text{off}} - \gamma a_1 \le 0$ because for $a_1 > a_2$, $A_+ < 0$, proving the lemma and showing that $a_{\text{switch}} = S_{\text{off}}/\gamma$. □

Note that the a_1 threshold for $f_1(S_{\text{off}}) = 0$ is independent of a_2. On the other hand, a decrease of a_2 causes $f_2(S_{\text{on}})$ to increase and $f_2'(S_{\text{on}})$ to decrease. Thus, depending on the other parameters, a_2 could induce a switch if it becomes small enough but this is not guaranteed.

In the singular limit, $u_i(t)$ immediately approaches $f(S_{\text{on}} - \gamma a_i)$ in the on-state and $f(S_{\text{off}} - \gamma a_i)$ in the off state. Hence, from (2) it will increase towards $f(S_{\text{on}} - \gamma a_i)$ in the on-state and relax back towards $f(S_{\text{off}} - \gamma a_i)$ in the off-state. It will have a net increase after each frame presentation cycle if the increase during the on-state is greater than the decrease during the off-state. We show that this is possible in the following lemma.

Lemma 2. *For $f(S_{\text{on}})$ large enough, which is achievable if f is nonsaturating and S_{on} is large enough, the local fatigue variable a_i can increase with each on-state until it exceeds a_{switch}*

Proof. We need to show that starting from a zero initial condition $a_1(t)$ can exceed $a_{\text{switch}} = S_{\text{off}}/\gamma$. The dynamics of the local fatigue variable a_i are given by (2). Over one period the net increase in a_i is

$$a_i(t+T) - a_i(t) = \frac{1}{\tau_a}\left(\langle u_i(t)\rangle - \langle a_i(t)\rangle\right) \ge \frac{1}{\tau_a}\left(\langle u_i(t)\rangle - a_{\text{switch}}T\right) \qquad (21)$$

where $\langle\cdot\rangle = \int_t^{t+T} \cdot\, dt'$. Thus, as long as $\langle u_i\rangle > a_{\text{switch}}T$ then $a_i(t)$ will increase with each on-state until it exceeds a_{switch}.

From (1) we obtain

$$\begin{aligned}\langle u_i\rangle &= -u_i(t+T) + u_i(t) + \langle f(S(t) - \gamma a_i)\rangle \\ &\ge -u_i(t+T) + u_i(t) + \langle f(S(t) - \gamma a_{\text{switch}})\rangle \qquad (22)\end{aligned}$$

and $\langle f(S(t) - \gamma a_{\text{switch}})\rangle = T_{\text{on}} f(S_{\text{on}} - \gamma a_{\text{switch}}) + T_{\text{off}} f(S_{\text{off}} - \gamma a_{\text{switch}})$. We now need to estimate $u_i(t) - u_i(t+T)$. For u_i dominant, the activity dynamics are

$$\frac{du_i}{dt'} = -u_i + f(S_{\text{on}} - \gamma a_i(t')),\ t \le t' \le t + T_{\text{on}} \tag{23}$$

$$\frac{du_i}{dt'} = -u_i + f(S_{\text{off}} - \gamma a_i(t')),\ t + T_{\text{off}} < t' \le t + T \tag{24}$$

Hence, $u_i(t)$ obeys

$$\begin{aligned} u_i(t+T_{\text{on}}) &= u_i(t)e^{-T_{\text{on}}} + \int_t^{t+T_{\text{on}}} e^{-(t+T_{\text{on}}-s)} f(S_{\text{on}} - \gamma a_i(s))ds \\ &\le u_i(t)e^{-T_{\text{on}}} + f(S_{\text{on}})(1 - e^{-T_{\text{on}}}) \\ u_i(t+T) &= u_i(t+T_{\text{on}})e^{-T_{\text{off}}} + \int_{t+T_{\text{on}}}^{t+T} e^{-(t+T-s)} f(S_{\text{off}} - \gamma a_i(s))ds \\ &\le u_i(t)e^{-T} + f(S_{\text{on}})(1 - e^{-T_{\text{on}}})e^{-T_{\text{off}}} + f(S_{\text{off}})(1 - e^{-T_{\text{off}}}) \end{aligned}$$

Yielding

$$\begin{aligned} u_i(t) - u_i(t+T) &\ge u_i(t)\left(1 - e^{-T}\right) - f(S_{\text{on}})(e^{-T_{\text{off}}} - e^{-T}) - f(S_{\text{off}})(1 - e^{-T_{\text{off}}}) \\ &\ge -(f(S_{\text{on}}) - f(S_{\text{off}}))(e^{-T_{\text{off}}} - e^{-T}) \end{aligned}$$

since $u_i(t) \ge f(S_{\text{off}})$. Thus, the condition for a_i to increase beyond a_{switch} is

$$\begin{aligned} \langle u_i\rangle \ge &-(f(S_{\text{on}}) - f(S_{\text{off}}))(1 - e^{-T_{\text{on}}})e^{-T_{\text{off}}} \\ &+ T_{\text{on}} f(S_{\text{on}} - \gamma a_{\text{switch}}) + T_{\text{off}} f(S_{\text{off}} - \gamma a_{\text{switch}}) \ge a_{\text{switch}} T \end{aligned}$$

However, since $a_{\text{switch}} = S_{\text{off}}/\gamma$ and $f(0) = 0$ this is

$$\langle u_i\rangle \ge -(f(S_{\text{on}}) - f(S_{\text{off}}))(1 - e^{-T_{\text{on}}})e^{-T_{\text{off}}} + T_{\text{on}} f(S_{\text{on}} - S_{\text{off}}) \ge a_{\text{switch}} T$$

By concavity $f(S_{\text{on}} - S_{\text{off}}) \ge f(S_{\text{on}}) - f(S_{\text{off}})$ and thus

$$\begin{aligned} \langle u_i\rangle &\ge (f(S_{\text{on}}) - f(S_{\text{off}}))(T_{\text{on}} - (1 - e^{-T_{\text{on}}})e^{-T_{\text{off}}}) \ge a_{\text{switch}} T \\ &\ge (f(S_{\text{on}}) - f(S_{\text{off}}))T_{\text{on}}(1 - e^{-T_{\text{off}}}) \ge a_{\text{switch}} T \end{aligned}$$

can be satisfied for $f(S_{\text{on}})$ sufficiently large. □

We now have all the pieces to establish when intermittent rivalry is possible. For intermittent rivalry, β must be sufficiently large such that the on-state is WTA. Consider the first epoch where $A_+ > 0$ (pool 1 is dominant) and both a_1 and a_2 are initially zero. Then by Lemmas 1 and 2, A_+ will become negative and dominance will switch. In the second epoch, pool 2 is dominant and 1 is suppressed. However, we can still use equation (19) by exchanging the indices. Epoch 2 differs from epoch 1 in that a_1 is now at a high value while a_2 is at a lower value. Thus a_1 will decrease while a_2 will increase. The switch will occur when $A_+ \leq 0^-$. This occurs when a_2 reaches the a_{switch}, which is guaranteed by Lemmas 1 and 2. Intermittent rivalry will then ensue.

We now consider the dependence of dominance time on frame presentation period. In the quartet illusion, T_{on} is fixed and T_{off} is changed. We obtain the following result.

Theorem 3. *Dominance time increases nonlinearly with T_{off}.*

Proof. The dominance time is given by the time it takes a_i to reach a_{switch}. During the on-state, u_i increases towards $f(S_{\text{on}} - \gamma a_i)$ and relaxes towards $f(S_{\text{off}} - \gamma a_i)$ during the off-state. In the singular limit, it attains its maximum $u_{\max}(t)$ at the end of the on-state, and its minimum $u_{\min}(t)$ at the end of the off-state. Similarly, a_i increases towards $u_{\max}(t)$ during the on-state and relaxes towards $u_{\min}(t)$ during the off-state. However, by Lemma 2, a_i will become progressively larger after each cycle. Let t be the time at the end of some off-state. Then after one frame presentation period, a_i has the value

$$a_i(t+T) = a_i(t)e^{-T} + \int_t^{t+T_{\text{on}}} e^{-(t+T-s)}u(s)ds + \int_{t+T_{\text{on}}}^{t+T} e^{-(t+T-s)}u(s)ds$$

which has derivative

$$\begin{aligned}\frac{da_i(t+T)}{dT_{\text{off}}} &= -a_i(t)e^{-T} - e^{-T_{\text{off}}}\int_t^{t+T_{\text{on}}} e^{-(t+T_{\text{on}}-s)}u(s)ds + u(t+T)\\ &\quad - \int_{t+T_{\text{on}}}^{t+T} e^{-(t+T-s)}u(s)ds\end{aligned} \tag{25}$$

$u(t)$ is decreasing during the off-state so its minimum is $u(t+T)$. Thus

$$\frac{da_i(t+T)}{dT_{\text{off}}} \leq -a_i(t)e^{-T} - e^{-T_{\text{off}}}\int_t^{t+T_{\text{on}}} e^{-(t+T_{\text{on}}-s)}u(s)ds + u(t+T)e^{-T_{\text{off}}}$$

which is always negative since $a_i > u(t-T)$. Hence, $a_i(t+T)$ will become smaller as T_{off} becomes larger. This then implies that it will take more frame presentation cycles to reach a_{switch} and thus increase the dominance time with increasing T_{off}. The increase will also be nonlinear (faster than exponential) in T_{off}. □

Remark 2. *The mechanism for intermittent rivalry could be considered to be release but it differs from release in static rivalry. Increased presentation frequency will induce faster switches because it causes a_i to increase faster. Reduced amplitude of pulses will also increase dominance period because it takes longer to reach threshold. Thus intermittent release is like escape for static rivalry, which implies Levelt's second proposition. It can also coexist with escape for static rivalry in the same model.*

Remark 3. *Similar arguments can be applied to show that intermittent rivalry is possible with cross-pool synaptic depression.*

We now consider the conditions that allow for slow habituation. Let the dominant and suppressed populations be labeled by D and S respectively. We show that the observed habituation of decreasing dominance times can occur if switches are always induced by the adaptation variable a_D reaching the threshold a_{switch}. The first epoch is caused by a_D reaching threshold starting from zero, and the second epoch is caused by a_D reaching the same threshold from a small number. Hence, the dominance times of the first and second epochs will always be similar. However, from the dynamics of (2) we see that the rate of increase in a_D slows linearly as a_D increases. Similarly, the rate of decrease of a_S also slows linearly as a_S decreases. Thus as long as the saturated value of a_D is greater than a_{switch} then a_D will reach threshold faster than a_S will decay to near zero. This implies that during the second epoch, a_S (which was the previously dominant population) does not decrease all the way to its initial value in the time it takes (the new) a_D to reach threshold. Thus, for the third epoch, the time it takes a_D to reach threshold will be shorter than the previous two epochs because it starts at a higher value. This will also occur for subsequent epochs and the decay value of a_S will get progressively larger and thus shorten the dominance time. This will eventually reach steady state where a_S is large enough such that it always relaxes back to the same state.

Now consider the case where there is depression but no adaptation. We again assume that population 1 is dominant. In this case, we can set $a_i = 0$

and obtain

$$2A_+ = f(S_{\text{on}}) - f(S_{\text{off}}) - \sqrt{\frac{f_1'(S_{\text{on}})s_2(T_n)}{f_2'(S_{\text{on}})s_1(T_n)}} f(S_{\text{on}} - s_2(T_n)\beta u_1^0) \tag{26}$$

In depression, s_1 decreases while s_2 increases, except for the first epoch if the initial conditions are $s_1 = s_2 = 1$. Increasing s_2 decreases $f_2(S_{\text{on}})$ while increasing $f_2'(S_{\text{on}})$ but because u_1^0 is very small, these changes are small. Hence, the main effect of s_2 is to increase the square root factor of the third term of (26) by its presence in the numerator. Conversely, decreasing s_1 increases this factor through the denominator. Hence, both of these processes serve to increase the third term and switching is possible for sufficiently strong depression. We can represent the switch condition, given by $A_+ = 0$, as

$$\sqrt{\frac{s_2(T_n)}{s_1(T_n)}} = \frac{f(S_{\text{on}}) - u_1^0}{f(S_{\text{on}} - s_2\beta u_1^0)} \sqrt{\frac{f'(S_{\text{on}} - s_2\beta u_1^0)}{f'(S_{\text{on}})}} \tag{27}$$

by rearranging (26). If $f(S_{\text{off}}) = u_1^0 << f(S_{\text{on}})$, $s_2 \leq 1$, $f'(S_{\text{on}})s_2\beta \geq 1$, $|f'(S_{\text{on}})/f(S_{\text{on}})| \sim O(1)$, $|f'(S_{\text{on}})/f''(S_{\text{on}})| \sim O(1)$, and $f''(S_{\text{on}}) < 0$, we can Taylor expand to obtain

$$\begin{aligned}\sqrt{\frac{s_2(T_n)}{s_1(T_n)}} &= \frac{f(S_{\text{on}}) - u_1^0}{f(S_{\text{on}}) - f'(S_{\text{on}})s_2\beta u_1^0} \sqrt{\frac{f'(S_{\text{on}}) - f''(S_{\text{on}})s_2\beta u_1^0}{f'(S_{\text{on}})}} \\ &= 1 + Cu_1^0 + O((u_1^0)^2) \end{aligned} \tag{28}$$

where $C > 0$ is $O(1)$.

In the first epoch, s_1 decreases from 1 while s_2 remains near 1. From (28), we see that dominance will switch when s_1 decreases to a threshold $\theta = 1 - 2Cu_1^0 + O((u_1^0)^2)$. In the second epoch, population 1 is now suppressed and s_1 will relax back to 1, while s_2 will begin to decrease from 1. The threshold condition is given by (28) but with the indices reversed. Since $s_1 = \theta$ is $2Cu_1^0$ less than 1, s_2 will take longer to decrease to threshold than s_1 in the first epoch. Hence, the dominance time of the second epoch will be longer than the first. In the third epoch, s_1 starts very near 1 (within much less than order u_1^0) and decreases while s_2 is order u_1^0 below 1 and increases towards 1. Hence, s_1 and s_2 will start in positions very near (much less than order u_1^0) from where they were in the second epoch. Thus, the dominance time will be almost the same as in epoch two and this will hold for subsequent epochs. These results are summarized in the following propositions.

Proposition 1. *Slow habituation occurs for local fatigue if the time constant is sufficiently long.*

Proposition 2. *Slow habituation will not arise for cross-pool depression.*
