# Supplementary figures and images for: Canonical Cortical Circuit Model Explains Rivalry, Intermittent Rivalry, and Rivalry Memory
Source: PLoS Comput Biol. 2016 May 3;12(5):e1004903. doi: 10.1371/journal.pcbi.1004903 (PMC4854419; doi:10.1371/journal.pcbi.1004903)

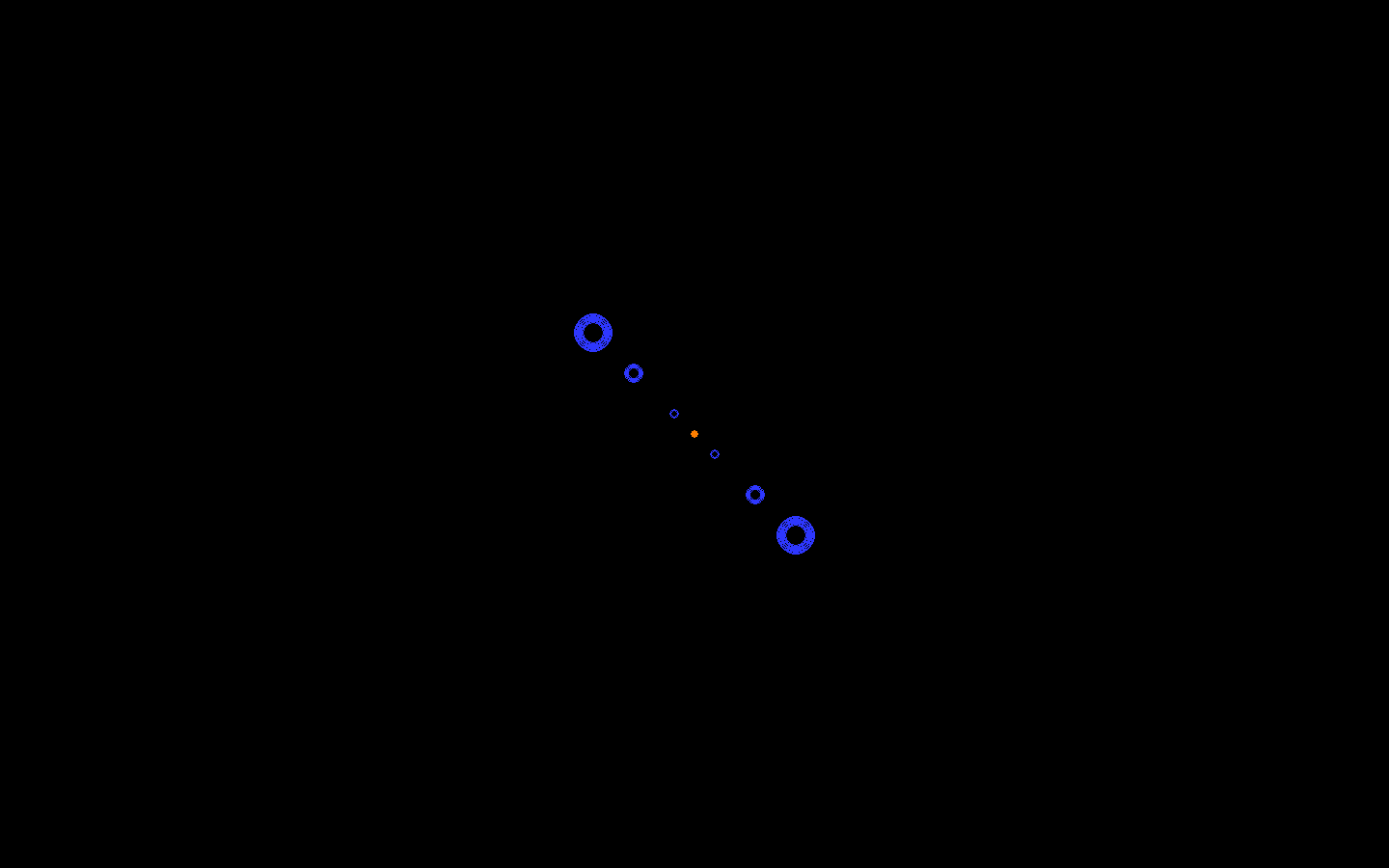

Supplement: S1 Movie — (ZIP) [file pcbi.1004903.s004.zip › stimulus/TF300.gif]
